# On the utility of Colour in shape analysis: An introduction to Colour science via palaeographical case studies

**DOI:** 10.1016/j.heliyon.2023.e20698

**Published:** 2023-10-10

**Authors:** Vlad Atanasiu, Peter Fornaro

**Affiliations:** a Department of Informatics, University of Fribourg, Boulevard de Pérolles 90, 1700, Fribourg, Switzerland; b Digital Humanities Lab, University of Basel, Spalenberg 65, 4051, Basel, Switzerland

**Keywords:** Colour science, Colour processing, Colour perception, Image processing, Image enhancement, Palaeography

## Abstract

In this article, we explore the use of colour for the analysis of shapes in digital images. We argue that colour can provide unique information that is not available from shape alone, and that familiarity with the interdisciplinary field of colour science is essential for unlocking the potential of colour. Within this perspective, we offer an illustrated overview of the colour-related aspects of image management and processing, perceptual psychology, and cultural studies, using for exemplary purposes case studies focused on computational palaeography. We also discuss the changing roles of colour in society and the sciences, and provide technical solutions for using digital colour effectively, highlighting the impact of human factors. The article concludes with an annotated bibliography. This work is a primer, and its intended readership are scholars and computer scientists unfamiliar with colour science.

## Highlights

• Did you know that you may improve shape analysis using colour information?
• This article introduces you to colour science and the image formation chain.
• It covers management, computational, psychological, and social colour aspects.
• You learn through practical illustrated case studies.
• Pursue the discovery of colour science using the article's annotated bibliography.

## Graphical abstract

**Figure:**
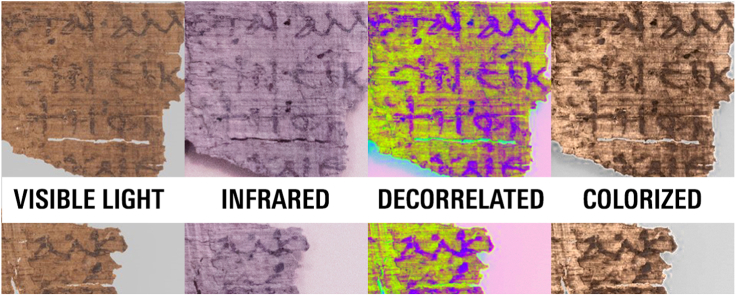

Whiteout, n. A white correction fluid used to cover textual errors.

— Oxford English Dictionary

## 1 Introduction

**Fig. 1:**
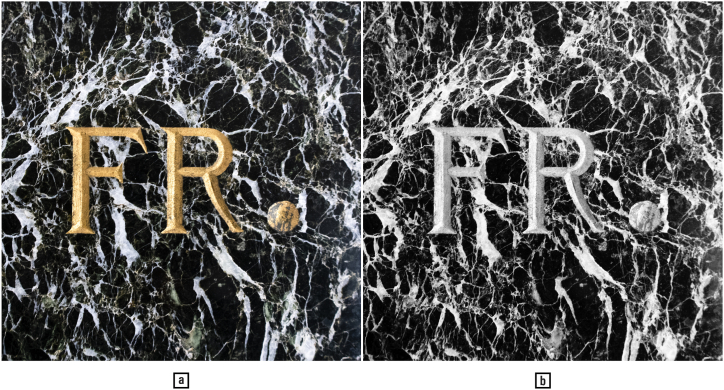
Colour (a) and grayscale detail (b) of the foundation inscription on the Cantonal, City and University Library Zürich, Switzerland. — Credits: Vlad Atanasiu.

With the passing of time, and without restoration, the gold-plated engraving on the marble on the left side of Fig. 1 may come to resemble the one on the right, pictured in grayscale. This example of natural camouflage and anthropic decamouflage illustrates the role of colour in alternatively enhancing and diminishing the legibility of letter shapes. Moreover, it is the special phenomenological quality of light reflected by gold — warm and immaterial, transcending shape, texture, and colour — that constitutes one of the essential aesthetic appeals of this type of baroque epigraphy. These effects can be experienced only in the full ambient physical reality comprising oneself, the inscription, and its material and spiritual surroundings. Thus, colours can also tell different stories about shapes than their outlines do.

In nature and visual media, phenomena pertaining to the interaction between colour and shape are pervasive. At a time of mass digitisation, digital imagery and computational methods supply novel and powerful solutions to unlock the potentialities of colour, while also making the mastery of colour science a necessity for scientific work, unlike what was customary during the analogue era, when not everybody was yet a photographer and a photoshopper. However, the interdisciplinary nature of colour science can make it difficult to be fully aware how a great variety of colour-related factors affect the capture, processing, display, and interpretation of shapes.

Informed by latent academic needs within a changing technological environment, the purpose of this article is to reach a readership new to colour science, in order to raise awareness of its benefits for shape analysis through an overview of the major aspects of theoretical and practical importance.∗

This article is addressed to scholars and computer vision scientists working in the fields of archaeology and cultural heritage who are unfamiliar with colour science, but may find that there are numerous benefits to considering colour and shape conjointly when analysing artefacts such as epigraphic writing, wall paintings, aerial photography, and three-dimensional digital models. In fact, it has been hypothesised that human colour vision first evolved to improve the species’ ability to detect and identify objects; in light of this fundamental function of colour, colour science becomes naturally relevant across a range of fields.

For illustrative purposes, this article focuses on the branch of historical sciences that is palaeography, as considered in its broad sense independent of writing substrate and instrument, and thus including such disciplines as epigraphy and papyrology. Palaeography is essentially the study of shapes, not colour; this is an advantage in the context of the present article, since it makes debating the merits of colour more challenging. It should, however, be relatively intuitive to transfer what is learned from the present investigation of palaeography to the study of shapes in other disciplines.

Considering the many interacting sub-divisions of colour science, this article is organized serially according to how much influence can be exerted by users on the various factors affecting the use of colour in shape analysis. This organisation also coincides with a progression from basic to advanced topics that need to be understood. The motivation for using colour in shape analysis is subject to the persuasion power of colour science advocates with respect to the expected benefits (Section 2); colour science aspects such as physics, imaging, management, models, and displays can be approached as prescriptive givens, while users may also contribute to their development (Section 3); computer science aspects are the most open to creative developments (Section 4); psychological factors may be beneficially leveraged and influenced (Section 5); finally, social aspects are the least influenceable, but it is necessary to be aware of them in order to adopt appropriate attitudes (Section 6). Each topic is introduced by framing it in terms of colour utility in shape analysis, and illustrated by one or more case studies, supported by visual explanations. A synthetic overview of the main lessons is provided in the conclusions (Section 7). The extensive and annotated bibliography provides avenues for readers to pursue their interests beyond this article.

There is no prerequisite to understand the following exposition, other than a willingness to engage in transdisciplinarity. The text draws mainly on an eclectic mix of visual psychology and image processing, with abundant cultural and historical references.

To create the right expectations about the nature of this work, we note here that it mixes elements of a *manifesto* programmatically (the exhortation to use colour), an *essay* stylistically (not a mechanistic proof), a *primer* content-wise (instructions for how to do things), a *survey* structurally (the catalogue of case studies), and *infographics* methodologically (visual illustrations). This multifaceted approach, similar to a *guided tour* showcasing the ‘Wunderkammer’ of colour, distinguishes it from typical research, review, and position papers. This approach was adopted to achieve our educational goals of ‘raising awareness’ and ‘introducing colour science’ in an accessible and comprehensive manner.

One strength of this article is that it introduces readers to a broadly relevant and timely shape analysis methodology that goes beyond the mainstream. In comparison to general-purpose handbooks and topic-focused articles on colour science, this publication offers a synthetic overview of a highly heterogenous field, containing both theoretical fundamentals and practical application examples. It deliberately emphasises multidisciplinary factors in colour-based shape analysis (given their objective ubiquity in this field) and the challenges of cross-disciplinary research. In doing so, it contributes to knowledge transfer between colour science, computer science, and the humanities. Last but not least, the article presents original content, including an overview of the changing scientific attitudes on colour, chromatic thinking models in sciences, and chromatic metaphors of shape, along with a comparison between ‘manual’ photo editing and image processing via programming (with remarkable results), and custom-made visualisations of concepts.

The limitations of this article derive from its format and the topic itself. As a synthesis article, it can offer only a glimpse of a vast subject of which much remains to be discovered. As for colour, its psychophysical effects and its interactions with shape are numerous, difficult to control, and subject to objective imaging variation and reproduction limitations, as well as subjective variation between individuals and cultures. Furthermore, the information presented here is a snapshot of our evolving understanding of colour. All these aspects are discussed in dedicated sections on (for example) colour management and display, colour-blindness and subjective colours, gender differences, and cultural attitudes between chromophobia to chromomania. Nevertheless, colour represents a supplemental lever in the analysis of shape that beseeches to be used. Aside from its practical benefits, it is also a fascinating intellectual topic.

## 2 Motivations to use colour in script analysis

*Colour plays an important functional role within the document machinery as a device for improving readability and navigation. For the reproduction, processing, expertise, and display of written artefacts, this information should, in consequence, be preserved and utilized advantageously. Some rationales spanning these diverse tasks are presented below.*

**Fig. 2:**
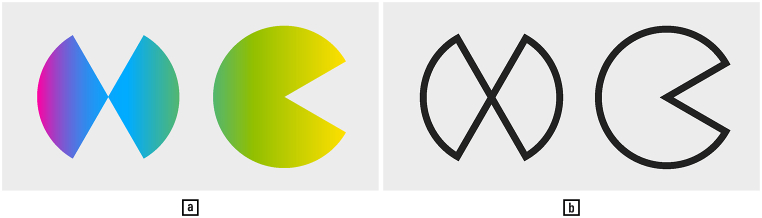
Polychromatic script (a) and its monochromatic variant (b). — Credits: Letters H and C, one resembling a butterfly and the other a Pac-Man, form the colour and outline versions of the logo of the Swiss Association ‘History and Computing’ (https://ahc-ch.ch), designed by Vlad Atanasiu.

One striking case for considering colour in shape analysis is that of polychromatic script surfaces (Fig. 2). Like butterflies, polychromatic scripts are examples of shapes that cannot be fully analysed or appreciated if colour is discarded. Alternatively, the script surface may be uniformly coloured, while multiple colours are employed for different symbols, words, or sentences, to make semantic distinctions and mark structural text entities. This technique is common in palaeography across cultures and periods. For example, the diacritics of the early Qurans were written in red, green, and yellow, while in one remarkable Maghrebine Quran, every other word changes colour; Persian Qurans, moreover, may contain interlinear Farsi translations distinguishable from the Arabic lines by being inked in red Ref. [[1]: pp. 115–157]. Gold may be used for the headings in Italian Renaissance manuscripts, or for the entire content of luxury medieval Qurans from North Africa and Buddhist sutras from Japan [[2]: 68–69, 110]. In contemporary Arabic-script calligraphy, polychromatic script surface characterises the signature style of Hassan Massoudy and his followers, who use writing instruments pigmented with multiple inks [3]. Today (here we transition from palaeography to ‘neography’, the study of contemporary script), coloured texts have become an experimental subgenre in modern literature, and colour notation a versatile technique from music and diagrammatics to mathematics and electronics — while colour highlighters, sometimes fluorescent, are commonplace stationery items, that the reader of these lines might be using at this very instant (literature: example: [4]; literature: history: [5,6]; music: [7]; diagrammatics: [8]; mathematics: [9]). In brief, the proper colour reproduction and computational processing of all such documents is indispensable for their analysis.

The palaeographical utility of colour extends beyond lavish polychromatic documents, if we consider the informativeness of micro-chromatic variation on script surfaces. Because ink accumulates at the end of a stroke where the writing instrument is lifted, while distinctive furrows are left when two strokes cross, the study of chromatic variation may allow for the inference of ductus direction and sequence [10]. This colour-based information is useful more generally to expertise, for dating and localising scripts, and for establishing authorship. On banknotes, chromatic effects are exquisitely intricated with shapes and incorporated as security devices, including in holograms, the importance of colour being such that the overall note colour may even give the currency by metonymy its popular name, e.g., ‘greenback’ for ‘dollar’ [11]. Again, colour reproduction fidelity and colour processing are indispensable to make such operations possible and successful.

Through experience, epigraphists working on worn-out inscriptions are well aware that script exists only insofar as it can be distinguished from the writing substrate, a segmentation task facilitated perceptually and computationally by the surplus of information introduced by colour. The legibility of flaking Pompeii inscriptions, gravestone epitaphs covered by lichens, and script intermixed with papyri fibres can be greatly improved through the use of colour-based computational enhancement techniques, as we shall see shortly.

Faithful colour reproduction and colour-aware image processing are also necessary for the documentation of the conservation state of documents. From art-historical and museographical points of view, extensive research must yet be conducted before the chromatic experience obtained from originals can be effectively conveyed through reproductions. The reflection of gold, the shimmering of texture, and the transparency of ink, for example, are notoriously difficult to capture, display, and measure with today's technologies, despite forming core aspects of the phenomenology of script, i.e., the emotions and connotations evoked by contemplating the written surface.

## 3 Fundamentals for the effective use of colour in shape analysis

*The reproduction of documents in visible light (imaging) involves many technical aspects, such as lighting, camera optics and settings, and the use of reference rulers and colour patches, all of which need to be mastered to produce the kind of high-quality images necessary for fine palaeographical analysis. Since these techniques have been extensively studied in the pre-digital era, we will focus in this section on some aspects of digital colour management that are often overlooked by palaeographers and computer scientists unfamiliar with colour science.*

Specifically, we demonstrate the benefits of colour fidelity using the case of document fragment matching; we illustrate how the digital image file format affects the human perception of shapes, as well as the quality of their computational processing; we point out that the judicious choice of the colour space in which digital images are represented facilitates their processing; and we explore the perhaps more obscure phenomenon of legibility varying with the colour gamut.

To show how these quite different topics fit into a single perspective and how they relate to the computer science, psychology, and sociology aspects described in the other sections, we will begin with an overview of colour science as a complex research domain.

### 3.1 Understanding the complexity of colour science

*This section aims to show the complexity of image formation, the multidisciplinarity of colour science, and the mental nature of images. It provides the conceptual framework within which colour-based shape analysis is performed.*

Let us briefly recall the process by which the phenomena we perceive as colour and shape are formed, from the physical and material stage to imaging and digital images to the perception of visual stimuli. Illuminant bodies such as the sun, fireflies, and artificial lightbulbs emit electromagnetic waves of specific lengths, a narrow band of which can be perceived by the human visual system: this is ‘visible light’, in the range of approximately 390 nm–730 nm. These ‘rays’ of energy make contact with various materials — an ink-inscribed papyrus, for example — that modify the spectral power distribution of the reflected light by absorption, thereby giving rise to colours specific to objects under the given viewing conditions. The outgoing energy quanta — the photons — are captured by an imaging system, i.e., a light-sensitive device such as a photographic camera, which typically consists of optics, a photosensitive sensor, electronics, and software. The output is a digital file that can be further computationally processed, transmitted, stored, and then presented on a display or printed on paper. Those operations, which involve the preservation of colour consistency, constitute the so-called colour management (practical: [12]; technical: [13,14]; ICC profiles: [15]; gamma: [16]). The results of this process are, first, a stimulus for the physiology of the eye, and thereafter for the neural activity of the brain experienced as ‘colour’. The interaction between colour and shape is interdependent on other perceptual features, such as transparency, shimmering, and texture, and other cognitive processes, such as memory, motricity, and language. These stimuli are interpreted according to individual, cultural, and social factors, and may prompt the viewer to take certain actions.

It is apparent that image formation is complex and involves many disciplines: physics, chemistry, optics, electronics, mathematics, computer science, psychology, neuroscience, philosophy, linguistics, history, and others. All these disciplines are part of colour science (compendia: [17,18,19]; data and formulae: [20]; history of colour science: [21]).

**Fig. 3:**
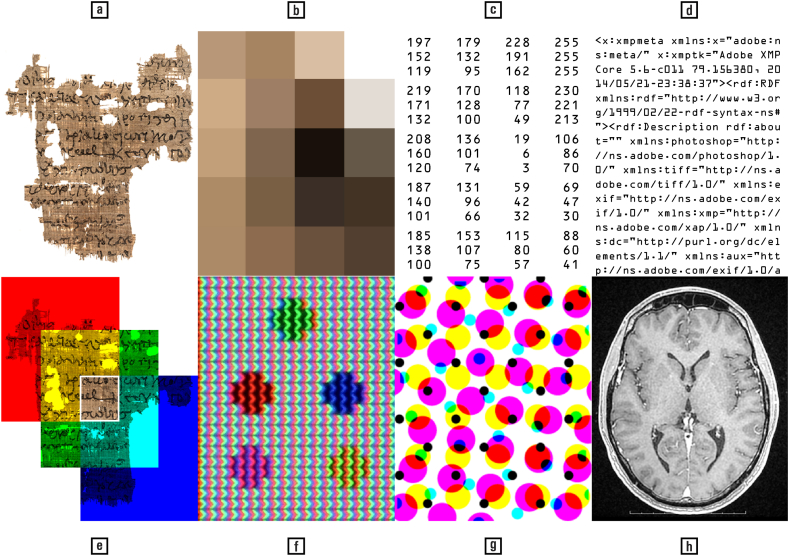
What are colours and shapes in a digital image, such as that of a papyrus (a)? Depending on what manifestation we are looking at, the answer is: (b) square colour pixels, which appear when zooming into an image; (c) a number matrix, defining the colour values of the pixels; (d) programming language, found by opening a XMP image metadata file with a text editor; (e) red, green, and blue colour channels that produce a colour stimulus via superposition; (f) a raster pattern of red, green, and blue filtered light emitters of a computer monitor, which fuses into five coloured dots when seen from far away; (g) the cyan, yellow, magenta, and black ink dots of a printed image, which also fuse into a pinkish uniform surface when seen from afar; (h) neural activity within the brain, experienced as an ‘image’. — Credits: a. P.Bas. inv. 47, Peter Fornaro, University of Basel Library; f, g. [26]; h. Vlad Atanasiu.

A further observation is that both colour and shape have radically different manifestations depending on their stage of formation. Fig. 3 presents some manifestations of a digital image: pixels, numbers, programming code, colour channels, liquid crystals, typographical dithering, and neural activity. By looking through a magnifier at a computer screen it becomes apparent how the shape of letters morph into rectangular dots, while the myriad hues dissolve into primary colours (red, green, and blue). In other words, what we call ‘colour’ and ‘shape’ exist only in our brains. These phenomena are illusions — sufficiently operational to keep us humans alive, but still illusions. They represent how we experience the world, not how it is. The perceptual interface theory, for example, explains this viewpoint by analogy with the icons on a computer desktop that facilitate interaction with the machine, but reveal little about its inner workings (psychology: [22]; philosophy: [[23]: 111–112]; nature of optical illusions: [[24]: xix–xxiii]; in Newton’s words: ‘For the [light] Rays to speak properly are not coloured.’ [[25]: 83]).

### 3.2 Colour management and data fidelity

*According to the op art artist Victor Vasarely* [[27]*: 990*]*, ‘form and colour are one and the same’. But if colours are not reproduced with fidelity, then the same object may appear as two distinct ones. The issue of faithful reproduction is at the core of ‘colour management’, as discussed in this section.*

**Fig. 4:**
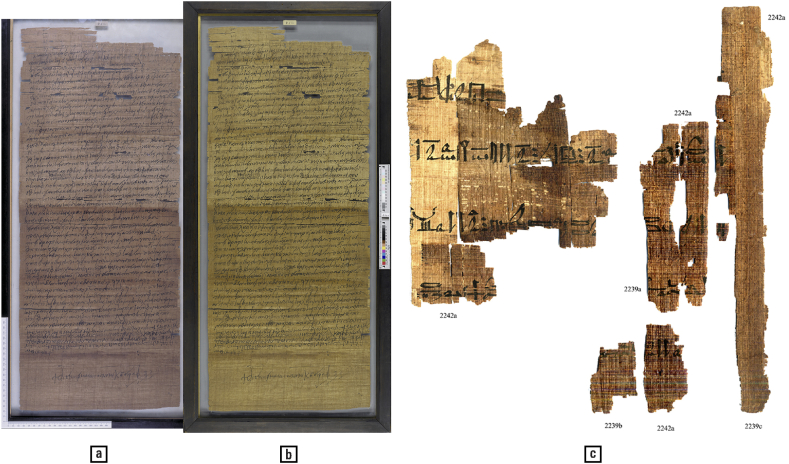
(a, b) Two reproductions of the same papyrus varying in colour characteristics. As neither has an embedded colour profile, it is only the first author of this article's memory of the original physical document that suggests (a) to be closer to the observed reality than (b). — A physically single document with (c) strong colour variation and (d) fragments attributed by expertise to the same document. — Credits: a, b. P. Hamb. graec. 175 recto, Staats-und Universitätsbibliothek Hamburg, https://resolver.sub.uni-hamburg.de/kitodo/HANSh795, (CC BY-SA 4.0); c, d. Inv.Sorb. 2239abc +2242a r, Florent Jacques, Institut de Papyrologie, Sorbonne Université, Paris.

Both natural and anthropogenic factors can cause the decomposition of documents into fragments. While their defragmentation may be desirable for various reasons, this is difficult to achieve without automation. Reasons for this can include the high number of fragments (e.g., in the well-known cases of the shredded documents of the former East German State Security organisation and those of the US Embassy in Iran in 1979 [[28]: 23, 34], or the worldwide dispersion of fragments between collections, as in the case of papyri). While colour is one of the document features that can aid in defragmentation, it is useful only insofar as colours are reproduced with fidelity. Fig. 4 presents images of the same document, taken by staff from the same institution at different moments in time (a, b). If these two images were different fragments of the same original document, it would be impossible to reconstruct the original based solely on colour information, because the reproductions are so different. This demonstrates the importance of proper quality control while digitising, as defined in digitisation guidelines such as the US Federal Agencies Digital Guidelines Initiative [29], ISO, and the Dutch ‘Metamorfoze’ [30]. When considering that the same document can exhibit significant and sudden colour variations (Fig. 4 c, d), it is evident that reproduction fidelity can help to avoid false fragment joins based on colour similarity. A stern enjoinment in this respect comes from physicians speaking about how, despite the importance of proper colour calibration in medical imagery — where boundaries of lesions, tumours, and other regions of interest may be less clearly defined as in palaeography —, the issue is too often ignored by practicians: ‘without reproducibility and accuracy of images, any attempt to measure colour or geometric properties is of little use’ [[31]: 2].

While good colour management is indispensable, it also has its limits. As the same authors observe (‘Taking two pictures of a wound with the same camera and settings, immediately after one another, normally results in two slightly different images’), the difficulty of determining shape boundaries is compounded by the fact that no two digital images of the same scene are objectively the same, due to environmental factors (e.g., change in illumination, object motion) and variations in imaging systems (e.g., different cameras, different settings, random electromagnetic noise) [[31]: 1, 2]. Image variability may be overcome in various ways. For example, electromagnetic noise in the camera can be reduced by averaging over multiple shots of the same scene, a technique known as ‘stacking’ commonly used in astrophysics [32]. Image variability may also be a desirable feature, as it reveals a host of information about the photographed scene and the imaging conditions [33].

### 3.3 Image formats and shape distortions

*While a number of sections in the present article explain how colour can improve shape processing and perception, this section illustrates the reverse case, i.e., how colour can negatively affect shape analysis.*

**Fig. 5:**
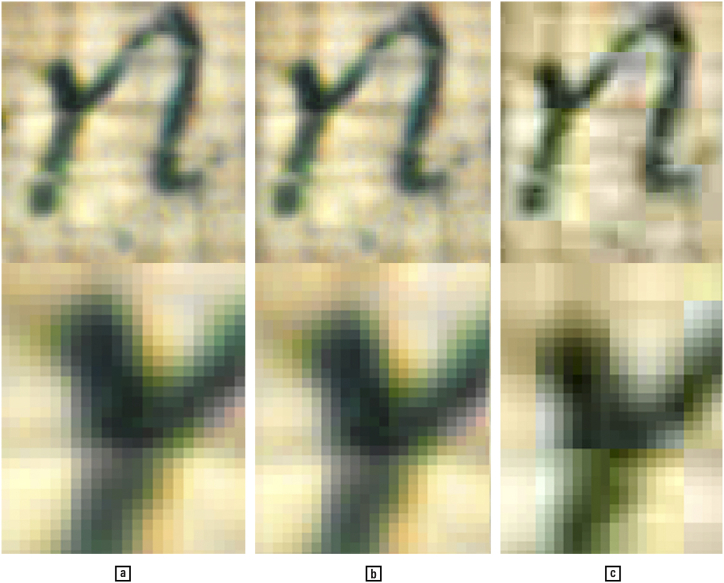
(a) Original TIFF format image, (b) converted to JPEG with 100 % quality and (c) 10 % quality.

Fig. 5 presents details of a papyrus at two zoom levels, two file formats, and two compression levels. There are almost no visible differences between the first and second image, even when zooming in, and from far enough away, the third image also appears similar; however, substantial ‘blocking’ and shape distortion become apparent at close range [34]. Such artefacts are obviously problematic for fine palaeographical analysis, both human and computational.

The phenomenon is partially due to rationales involving colour: because the size of image files can be considerable (723 Mb for the entire image from which the detail in Fig. 9 a was extracted), they can take up significant storage space and be slow to transmit over computer networks. The JPEG format was thus developed to reduce file size while maintaining good perceptual image quality (86.4 Mb and 5.8 Mb in Fig. 9 b and 9.c, respectively), originally for application in videotext. Presently it is one of the most common digital image formats for cameras and on the Internet. Two of the methods implemented in the JPEG format to achieve such compression leverage specific characteristics of human visual perception: namely, the lower sensitivity to the blue colour band, and the lower sensitivity to high-frequency chromatic information (i.e., small spatial variations in saturation) as opposed to high-frequency achromatic information (i.e., details distinguished by lightness) [[35]: 23–28; [17]: 26–29; [36]]). By rounding a parameterizable number of values associated with these features (a process called quantisation and performed in the frequency domain on eight-by-eight blocks of pixels), the JPEG file size is reduced. Other common image file formats, such as PNG, GIF, and RAW, have other particularities.

While JPEG artefacts also occur for greyscale images, the reason why they occur in the first place is due to technical and perceptual solutions involving colour, for which this image format was optimised. This section attempts to point out these pitfalls for the benefit of the reader. Additionally, it showcases how colour management can affect shape quality through the choice of image formats, and further highlights that while the resulting degradations might be invisible to the human eye, they may nevertheless adversely affect computational image processing.

### 3.4 Colour spaces and image processing ease and validity

*The way in which colour is described defines a ‘colour space’. Because colour spaces are designed for specific applications, a large and increasing number of such spaces exist [* 37 *,* 38 *]. Here, we present some of the more prevalent types, along with their impact on colour and shape analysis, as exemplified by the colour distribution of a papyrus image (* Fig. 5 *).*

An RGB-based colour space defines colours in terms of the mix of the primary colours — red, green, and blue — that are necessary to obtain a given colour on most common displays. The display primaries are derived from the physiology of the human eye, which contains around seven million cells — ‘cones’ — that are individually sensitive to one of three overlapping ranges of light wavelengths (hence the term ‘trichromatic’ to characterise ‘normal’ human vision), and whose combined output is eventually perceived as colour [39]. Typical photographic cameras, file formats, and displays reproduce this architecture, which works as a biologically inspired means of capturing, storing, and transmitting colour. (Under low light conditions cones are insensitive, while around 120 million ‘rod’ cells become responsible for vision, and their activity results in achromatic perception.)

L*a*b* (or CIELAB) is another colour space, based on psychophysical research dating back to the 1920s and carried out by the International Commission on Illumination (CIE), the leading standardisation organisation in colour matters, with the aim of characterising the perceptual qualities of colour. The orthogonal dimensions of L*a*b* are ‘lightness’ (L*, defining the black–grey–white achromatic axis), ‘green–red’ (a*), and ‘blue–yellow’ (b*; the two chromatic axes), and those of its cylindrical LChH version are ‘lightness’, ‘chroma’ (similar to ‘saturation’, and perpendicular to lightness), and ‘hue’ (circular around lightness) [[37]: 85–96]; these are not only more immediate and complete colour descriptors for humans than red, green, and blue, but also take the mathematical non-linearity of human colour perception into account. Additionally, using L*a*b* or LChH makes certain types of colour arithmetic more straightforward. For example, segmenting objects from their background according to their lightness level is simpler in L*a*b*, where lightness is one of the dimensions, than in RGB, where an approximation of lightness is given by the diagonal of the distribution cube. Moreover, the lightness of an image, which is useful in many computational applications, can be trivially obtained in the L*a*b* colour space by setting the a* and b* channels to zero.

**Fig. 6:**
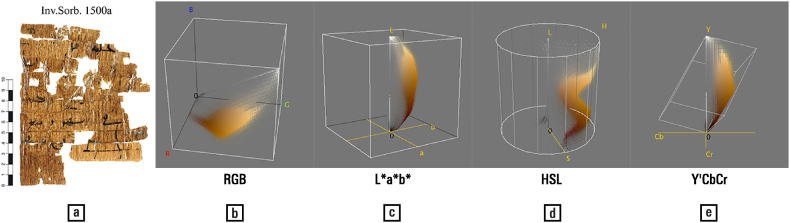
(a) Papyrus image and (b–e) its colour distribution in various colour spaces. — Credits: papyrus: Inv.Sorb. 1500a, Florent Jacques, Institut de Papyrologie, Sorbonne Université, Paris; visualization: Colour Inspector 3D and ImageJ.

The HSL colour space uses linear transformations of the display system R, G, and B values to encode colour as an approximation of hue, saturation, and lightness, and has gained popularity among early computer graphics researchers due to its intuitive colour representation; however, it has poor psychophysical validity. Colour differences, for example, are not perceptually uniform, as intended in the L*a*b* colour-space, which is obtained by non-linear mathematical transformations [[35]: 20]. Furthermore, colour distributions can acquire complex shapes, as presented in Fig. 6, and thus become difficult to manipulate mathematically.

The Y'CbCr colour space — where Y' stands for ‘luma’, and Cb and Cr for ‘chroma’, these descriptors being representative of lightness and chromaticity, respectively — was developed for television broadcasting and has been adopted for video production [40]. These are both applications in which signal transmission speed (or ‘bandwidth’) is critical, resulting in a reduction of the perceptually secondary chroma versus a full-quality luma. The sampling process is visible from the striped appearance of the distribution.

Note how the above colour spaces are limited to hue, saturation, brightness, while colour is characterized by many more effects: transparency, sheen, glitter, colour interactions, and so forth. These are considered in more sophisticated ‘colour appearance models’ (CAM) [37].

### 3.5 Colour gamut and shape discriminance

*The volume and topology of the space of perceived colours determine shape discriminance.*

**Fig. 7:**
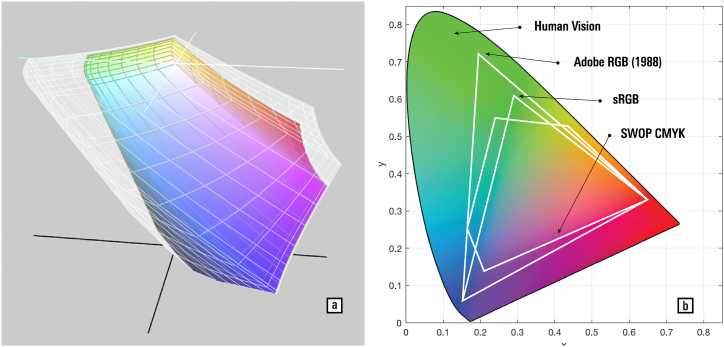
(a) The coloured solid represents the sRGB colour gamut, the most common profile of digital images used by photographic cameras and exchanged over the Internet, within the wider Adobe RGB (1998) gamut (greyed solid), which is a richer gamut intended for higher photographic quality. This is a screenshot of ColoSynch, a colour profile selection tool included in Macintosh computers. (b) A cut through the CIE XYZ colour space showing the extent of four types of colour gamuts: human vision, Adobe RGB (1998), sRGB, and SWOP (Specifications for Web Offset Publications).

Natural and artificial imaging and reproduction systems, such as eyes, cameras, and ink, are limited regarding the number of different colours to which they are sensitive, or that they can render; moreover, colourblind people are insensitive to some colours, while all of us perceive colours slightly differently, even if we are unaware of these phenomenological differences (in other words, roses are red for everybody in name alone) (on gender-based hue shift, see Refs. [41,42]). The amount of sensed or rendered colours is called a ‘colour gamut’, which defines a specific volume size and shape in a colour space. The larger the gamut, the more numerous, vivid, and pure will be the colours that can be sensed or rendered, with human vision having a vastly larger colour gamut than can be produced in print or on displays (Fig. 7). Colour gamuts also form part of the ‘colour profiles’ used to describe the overall colour characteristics of imaging and display devices, and allow conversion between them, using so-called Profile Connection Spaces [15]. Knowledge of the profile allows these devices to attribute the numerical values found in digital image files to specific colours of the gamut, and, as a consequence, in the physical world.

**Fig. 8:**
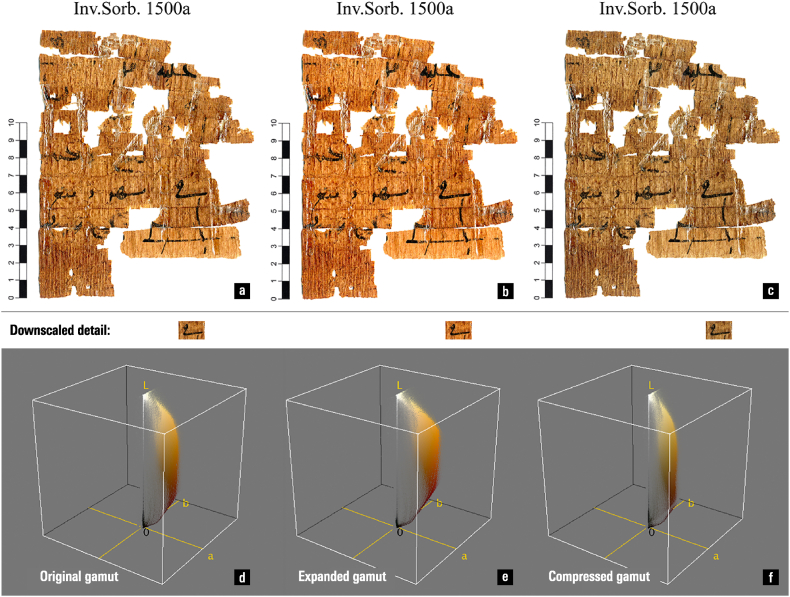
A papyrus (a) imaged with the sRGB colour profile, (b) opened with the Adobe RGB (1998) colour profile, and (c) converted back to sRGB. Note the changes in saturation, colour contrast, and legibility. The image (b) has the largest gamut, strongest contrast, and best legibility; the effect is stronger for finer details, or when looking at the target from farther away. (d–f) The colour distribution of the papyrus images in the L*a*b* colour space. The shape in (f) has the largest extent, and the one in (g) the smallest. — To reproduce these images, follow the procedure outlined here [28]: The software used is Photoshop CS6; conversions use the Adobe ACE Conversion Engine and the Perceptual intent; files are saved to TIFF format, with embedded colour profile. (a) File > Open > select Assign profile: sRGB; Edit > Convert to Profile: RGB: sRGB; File > Save As; (b) File > Open > Assign profile: Adobe RGB (1988) > Convert to sRGB > Save; (c) File > Open > Assign profile: sRGB > Convert to Profile: Adobe RGB (1988) > Assign sRGB > Save. The colour distribution visualization is created with the Colour Inspector 3D plugin in the ImageJ software package. — Credits: a–c. image: Inv.Sorb. 1500a, Florent Jacques, Institut de Papyrologie, Sorbonne Université, Paris; image processing: Vlad Atanasiu.

To maintain colour fidelity, the imaging gamut should be smaller or equal to the reproduction gamut, although this rule can sometimes be broken to produce specific effects. Fig. 8 illustrates an improvement in the legibility of the papyrus achieved by converting an sRGB gamut image to the Adobe RGB (1998) gamut. The phenomenon occurs due to an increase in saturation, which acts as a contrast enhancement in the colour domain at near-identical lightness distribution.

**Fig. 9:**
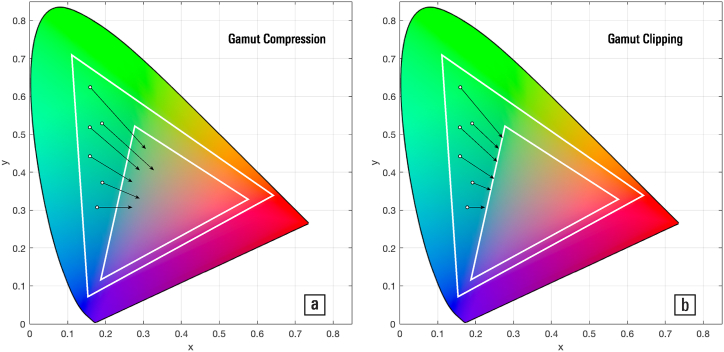
Gamut mapping by (a) linear compression and (b) clipping (after [133: 34]).

### 3.6 Gamut mapping and cross-media colour constancy

*We have seen that the colour gamut is a limited volume in the colour space and that different imaging and display systems have different gamut characteristics. Let us now consider what happens to images and shapes when they are transferred between distinct colour systems.*

When the gamut of an image is larger than that of the target display, the gamut is shrunk to avoid the presence of colours that cannot be reproduced — this, and the converse operation of gamut expansion, is called ‘gamut mapping’ [43]. Intensive research has been carried out on how to best convert between gamuts for various applications. Fig. 9 illustrates shrinkage by (a) linear compression and (b) clamping out-of-bounds colours to the outer envelope of the target gamut. The first algorithm maintains the relative perceptual difference between colours, but reduces the overall saturation of the image, making it look less vivid and thus inadequate for applications where saturation is required (such as advertising). The second algorithm would preserve the colour appearance of the image if there were few out-of-bounds colours, but would drastically degrade its appearance if most colours were out of bounds.

A number of observations can be made at this point. First, the choice of gamut mapping algorithm, and implicitly its impact on shape integrity, is not a purely mathematical issue, but rather depends on both data characteristics and usage intent. Colour management applications typically allow users to select between methods for maintaining the fidelity of overall colour perception, saturation, and numerical values.

Second, gamut mapping is often opaque, insofar as users can only select mappings by choosing from terms denoting general ‘intents’, which provide no information about how they are implemented algorithmically. These methods can and often do vary between software programmes, if not also within the same application. In Photoshop, for example, the difference between ‘Assign Profile’ and ‘Convert to Profile’ is not explicit; depending on which is used and how they are combined, the results can be inconsistent or perplexing.

Third, gamut mapping, with all the uncertainty and complexities involved, can take place without users being necessarily aware, by simply opening and saving a digital image file. For example, this occurs if the image has no colour profile specified, meaning that the software must attribute one by default in order to process the content (in such a case, the sRGB colour profile is typically assumed).

## 4 Methods and applications for computational colour-based shape analysis

*The digital enhancement of images to improve legibility by humans and processing by machines, along with the segmentation of text from background, are routine and elementary stages of palaeographical work during which colour is often discarded and the processes are performed on grayscale images. This section illustrates how the promoted methodology of taking colour into account may yield improved results for these basic shape-related tasks by automated and manual means alike.*

### 4.1 Colour-based image enhancement

*Dropping colour information alters the ability to discriminate shapes – and vice-versa.*

Two essential tasks carried out by members of the humanities disciplines in connection with documents are to read and publish them. Both of these tasks benefit from image legibility; thus, legibility enhancement is a valuable computational research area for the editing of texts. The challenge is to selectively enhance script rather than other structures while retaining sufficient non-script information to avoid misinterpretations and enable discoveries.

**Fig. 10:**
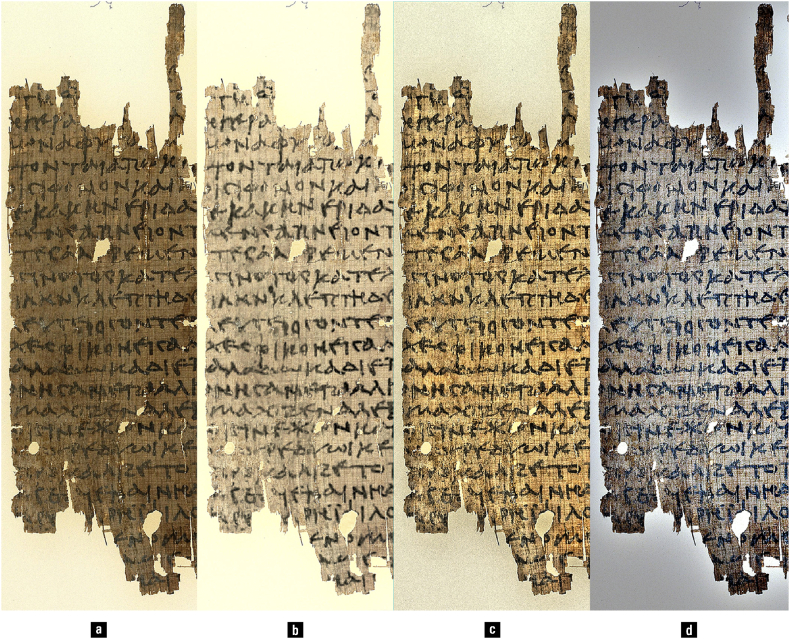
(a) Original papyrus image; (b) enhancement combining information across different colour spaces; (c) contrast-limited adaptive histogram equalisation (CLAHE) of the image's achromatic channel; (d) retinex-based enhancement. — Credits: P.Mich.inv. 1318v, University of Michigan Library; image processing by Vlad Atanasiu.

Fig. 10 illustrates the impact of various colour- and non-colour-based image enhancement techniques on legibility. The comparison indicates that the addition of colour in the image processing procedure may lead to increased legibility (images b and d vs. image c), and that not all colour-based enhancement methods are equal (image b vs. d). Some of the other insights obtained from this specific figure also have general relevance. For example, typical papyrus images found in online databases often have low contrast and are thus difficult to read (a). Contrast-limited adaptive histogram equalisation (CLAHE) of the image's achromatic channel (i.e., lightness) visibly improves legibility, but also enhances the script and the papyrus texture in equal measure, creating a confusing interference between the two (c) [44]. By combining information across different colour spaces, better legibility may be achieved through selective script enhancement, background equalisation, and preservation of input information at low-intensity levels (b) [45]. Moreover, the fact that the technique applied here was developed by the first author specifically for papyri enhancement cautions against the uncritical use of generic enhancement methods. As a case in point, the illustrious retinex theory, developed by Edwin H. Land, the inventor of the Polaroid camera, makes sophisticated use of colour, and results in strong local contrast, although it also produces an undifferentiated enhancement of all papyrus structures, sometimes in addition to a darkening halo effect (d) (original theory: [46]; algorithm families: [47]; development examples: [48,49]). In conclusion, no single method is sufficient for all documents and applications.

### 4.2 Multispectral imaging and revealing invisible shapes

*Light beyond the visible spectrum may reveal otherwise-hidden shapes.*

The human visual system is sensitive only to three colour components, perceived as red, green and blue, which correspond to electromagnetic waves with peaks at 645 nm, 526 nm, and 444 nm, respectively. The full range of colours, with various wavelengths, can be generated through a linear combination of these signals. Due to the limitation of these three sensitivities, an infinite set of spectral power distributions exist that we perceive as the same colour. One example is the addition of red, green, and blue, on one hand, and cyan, magenta, and yellow, on the other hand, which both result in the perception of white. The phenomenon is called metamerism and characterises the human visual system, as well as all technical devices based on three colour components [[37]: 335–337; [39]]. While the confusion caused by metamerism may appear as an impediment to the survival of biological organism, its impact is mitigated by the low relative frequency of the phenomenon in natural scenes [50], and it forms, moreover, the basis of efficient colour vision. However, metamerism remains an impediment for the objective description of physical properties, such as ink. To overcome this limitation, capturing visual signals with more than three components is a state-of-the-art solution, known as multispectral imaging. By adding layers of non-visible wavelengths to an image, specific material properties of artefacts can also be taken into account. For example, certain chemical binders react strongly with ultraviolet light. Due to their material and chemical properties, the contrast between script and substrate can vary along the spectrum, resulting in better legibility in a specific band or mix of bands [51,52]. The science and art of the multispectral approach therefore consist in a combination of multispectral imaging, multispectral image processing, and multispectral image representation and interaction.

**Fig. 11:**
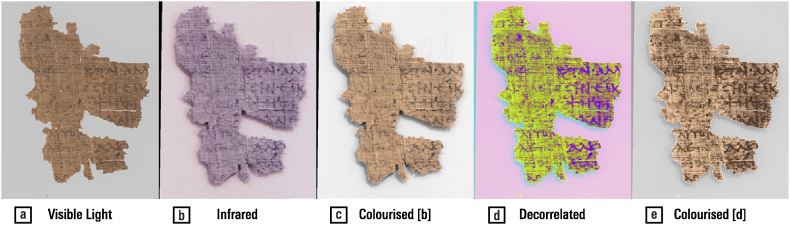
Multispectral images of a papyrus inscribed with iron-gall ink: (a) visible light; (b) infrared; (c) colourised infrared lightness with visible light saturation and hue; (d) decorrelated red visible light, blue visible light, and infrared lightness; (e) colourisation of (d). — Credits: a. P.Bas. inv 27, University of Basel Library; b. Peter Fornaro; c–e. Vlad Atanasiu.

Figure 11 reveals the differences in a papyrus inscribed with iron-gall ink, as it appears after the mixing of the wavelengths perceived as red, green, and blue (a) and in infrared, at 740 nm (b). Note that legibility can be improved simply by colouring the lightness component of the infrared image with the saturation and hue of the visible-light image, without the need for additional complex procedures, making the infrared image appear more familiar in terms of colour (c). Much better results are obtained by applying the mathematical procedure of decorrelation to the infrared lightness and visible-light red and blue bands, a process that effectively fuses pieces of a script from visible-light and infrared (d) [[53], [54], [55]]; colourisation of the result might also be preferred by some viewers (e).

One practical conclusion that can be drawn from this discussion on multispectral image processing concerns the power of combining existing methods to improve overall performance. In the following sections, the reader will discover more examples of this technique.

### 4.3 Colour-based shape segmentation

*In black and white, it is difficult to distinguish shapes that may be trivial to separate in colour vision.*

**Fig. 12:**
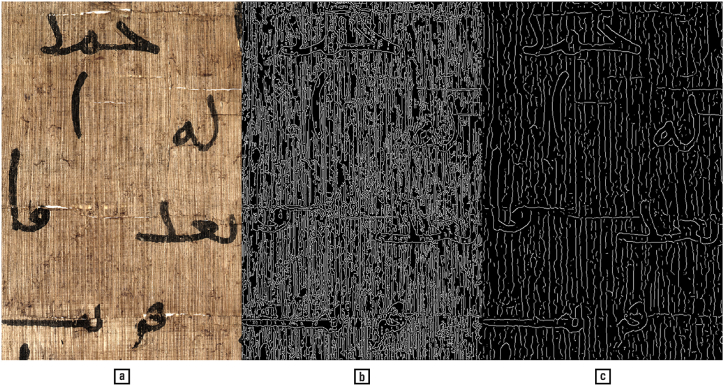
(a) Colour image of a papyrus. (b) Edge detection using the Canny method for the lightness gradient [59]. (c) Applying the same method on the colour gradient. — Credits: a: Inv.Sorb. 2346, Florent Jacques, Institut de Papyrologie, Sorbonne Université, Paris.

It is notoriously difficult to segment script from background in papyri documents, as their textured surface produces shadows of similar intensity to the script (see examples from a papyri binarization competition in Ref. [56]). However, better results can be obtained by performing the segmentation in the colour domain (Fig. 12), which provides additional useful information, i.e., saturation and hue [57]. The outcome may be further improved by integrating data from multiple features, such as colour and texture [58].

### 4.4 Colour-based background removal

*Even apparently monochromatic shadows contain traces of colour and may thus benefit from colour processing.*

**Fig. 13:**
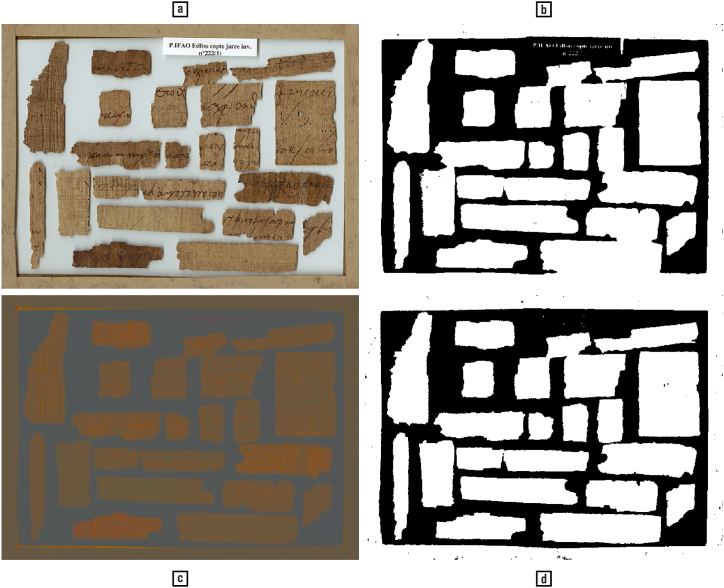
(a) Colour image of papyri fragments; (b) binarization of image ‘a’ using the popular Otsu method; (c) colour-invariant version of image ‘a’; (d) binarization of image ‘c’. — Credits: Institut français d'archéologie orientale (Ifao), Cairo; d-scribes project, University of Basel; ‘Edfou au VII^e^ siècle’ project, Ifao.

To further showcase the utility of computational colour image processing, we present the task illustrated in Fig. 13, which consists in segmenting objects from the background and from each other (a). To this end, the image was, in one case, reduced to lightness and binarized using the popular Otsu method (b) [60]; in the second case, the shadows were removed by taking all colour information in the red, green, and blue channels into account, thereby producing a colour-invariant image [[61]: 50–51] (c) that is subsequently binarized (d).

Biological systems also have demonstrably more difficulty suppressing shadows in the lightness channel alone than in all channels concomitantly [62]. Consider that a colour-edge usually characterises a change in material, while a lightness-edge is ambiguous: it may be either a change in material or the edge produced by a shadow (the latter phenomenon is used for camouflage) [63,23]. In this case, too, colour improves a purely shape-oriented task, as it is the source of a different kind of information about our surroundings. The utility of colour in our experience of the world as stable under a wide range of changing illumination conditions — named ‘lightness’ and ‘colour constancy’ [64] — rather than a perpetual kaleidoscopic composition and decomposition of shapes has been invoked to explain the emergence of colour vision itself [[[65], [66]]: 224–225].

### 4.5 Integrating human and machine abilities

*Up to this point, we have championed the utility of automated methods for colour-based image processing — but what about interactive photo editing? Can the human outperform the machine? In which contexts and for which reasons?*

**Fig. 14:**
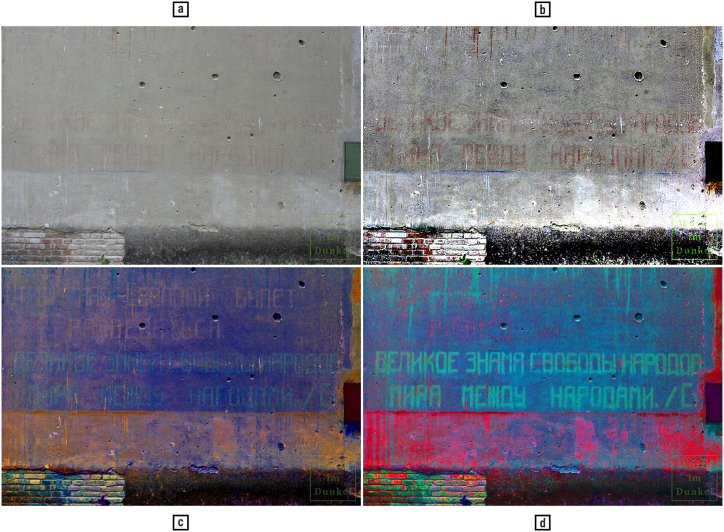
(a) Unprocessed image; (b) image processed in ImageJ with one implementation of the retinex approach, which gave the best results in terms of script legibility among those tested by the authors; (c, d) image edited in Adobe Photoshop by an expert in digital inscription recovery [69]. The modified pictures are processed on the three colour channels of the input RGB image. Note that the top two lines of text are almost invisible in both the unprocessed and the retinex versions. — Credits: Russian inscription from 1945 on a mansion in Lower Austria, announcing the end of the Second World War; photographed and processed by Thomas Keplinger, https://www.worteimdunkel.at/?p=6713.

The post-processing of historical images (e.g., for restoration, colouring, colour balance and superresolution) is a domain of predilection for photo-editing, sometimes with stunning results (see Ref. [67] for colouring famous black-and-white pictures, and [68] for superresolution images from NASA's Apollo programme). As Fig. 14 demonstrates, humans can achieve superior results on specific images and tasks, provided that they possess the right skills. The reasons behind this disparity include the flexible integration of various methods into workflows, know-how relative to their sequencing and parametrisation gained through lengthy experimentation, the specific enhancement of different image regions beyond the capabilities of existing locally adaptive methods, contextual semantic knowledge, application-specific reasoning, and the cognitive ability to perform effective image quality assessment.

Some of these aspects, taken individually, have been thoroughly studied in image processing (compendium: [70]), and the desire for their integration has led to the development of methods fusion research [71]. Other aspects, such as binarization, remain challenging despite decades of research [45]. Faced with certain limits of automated image processing when confronted with real-world applications (document enhancement is one example), and realising their complementarity with the human aptitude for interpreting visual stimuli, researchers have progressively established the interdisciplinary field of perceptual image processing. Examples of research directions in this field include the coupling of image enhancement and image quality assessment [72], along with the systematic exploration of leveraging perceptual phenomena for the purpose of image enhancement [73,74]. Human colour perception and computational processing is a further example, as discussed throughout this article.

It remains — and this is the ‘lesson’ of this section — that image processing is both science and art, especially when colour is involved.

## 5 Leveraging psychological factors for colour-based shape analysis

*When using colour in the analysis of script, the palaeographer must contend with a host of effects that affect the perception of colour and shape in both beneficial and detrimental ways. The computer scientist, too, should be aware of such human factors insofar as the computational methods to be developed need to match the specificities of the human visual system. Moreover, synchronic and diachronic cultural variability in colour perception and naming and in colour-related technologies must also be accounted for in machine learning and automated shape classification. The case studies discussed in this section should make these points clear.*

### 5.1 Utility of colour vision: past and present

*A look at the prehistorical origins of colour vision illuminates contemporary phenomena related to the use and neglect of colour in shape analysis, particularly as affected by gender differences.*

**Fig. 15:**
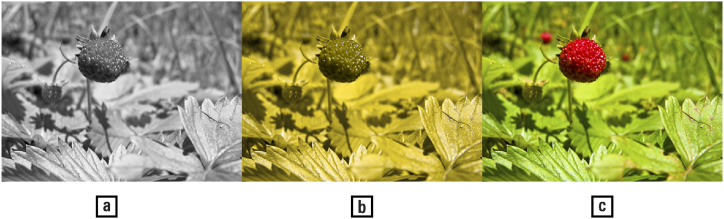
(a) How many ripe berries will you eat at breakfast using monochromatic vision? Answer: perhaps one. (b) Using dichromatic vision? Also one. (c) And using trichromatic vision? At least three! — Credits: picture: Vlad Atanasiu; berries: Lower Austria.

Exactly why animals developed colour vision is unclear. The extraction of information about the environment from a spectral analysis of light wave frequencies, amplitudes, and phases requires multiple photoreceptors, and the trichromatic architecture of ‘opponent colours’ provides an efficient and compact mechanism for this purpose [[75]: 14–18; [37]: 22]. Clear benefits for trichromats are a reduction in the ambiguity induced by shadows in respect to shape detection and identification (i.e., improve ‘colour constancy’). But what do mantis shrimps do with up to fifteen types of photoreceptors, including sensitivity to ultraviolet and circularly polarized light [[76]: 35, 42–44, 235–237]? For the colour vision of primates there are various explanatory theories, mostly argued in terms of statistical properties of the visual environment, involving the detection of ripe fruits in thick foliage and avoidance of toxic plants, the primates' visual sensitivity peeking in the green-yellow band of light (Fig. 15) [[21]: 198]. This is perhaps a mechanism for the co-evolution of flora and fauna: the animals helping the spread of seeds, in a similar manner to flower-bearing plants encouraging pollination by insects attuned to the ultraviolet colour of their petals, to the point that their reliance for navigation and feeding may be even greater on colour vision than shape discriminance (overviews: [[[77], [78], [79]]: 112–119]; ecological factors: [80]; fruit theory: [81,82]; ultraviolet vision: [83]).

**Fig. 16:**
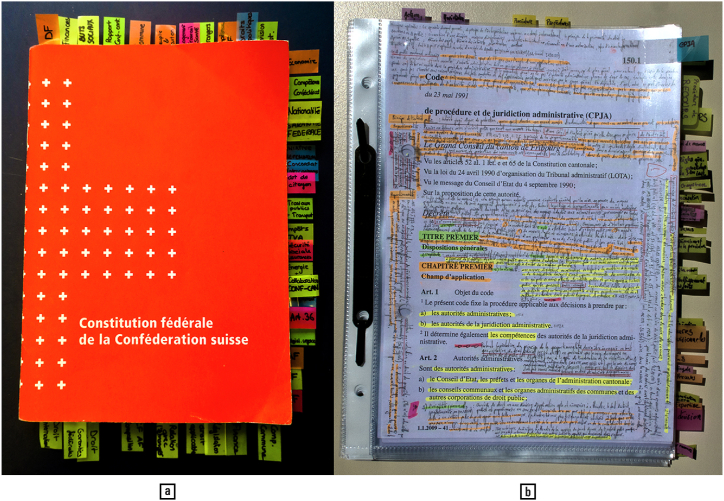
Colour vision and academic achievement: (a) colour-coded bookmarking and (b) highlighting by university students. Note that the number of hues used is higher than the basic four opponent colours, one female being observed using a twenty-three-colours highlighter megapack. From an ergonomics perspective, there are a few remarkable points suggested by these images: some tasks and methods are apparently more effectively approached using colour (structuring a black-and-white document), some individuals and groups are more able to discriminate between closely related colours, and the benefits of using colour are greater than the effort of managing a large array of colouring tools. — Credits: Vlad Atanasiu.

As urban humans no longer need to pick berries for survival, they may afford a higher amount of colourblindness than rural dwellers. Urban humans, however, still benefit from colour vision for intellectual work. The pictures in Fig. 16 show the extensive usage of colour for bookmarking and highlighting documents, as practiced by law students at the University of Fribourg, Switzerland. These markings make the documents browsable for navigational purposes on the basis of information encoded in colour alone, just as riffling green leaves to look for red berries. They also question the notion that outlines are sufficient for object recognition [[75]: 8, 121–138].

Remarkably, the importance of colour for safety has persisted in the transition from the natural to the human-made environment, even becoming ubiquitous in conjunction with shape-based markers (text, symbols, and patterns) applied on objects in areas as diverse as pharmaceutics (distinguishing drugs in hospitals [84]), transportation (the yellow school bus), construction (green fluorescent jackets), and electronics (colour-coded specifications).

In general, the number of colours used in drawing tasks is higher for females than males [85], reflecting a broader spectrum of gender-related evolutionary characteristics of female chromatic perception, such as faster, richer, and more accurate colour naming ability [[86]: 320], better discrimination in the middle of the visible light spectrum than males [41], as well as higher rates of being genetically potential tetrachromats [[87]: 200, 206; [88]], and thus endowed with finer chromatic sensitivity.

**Fig. 17:**
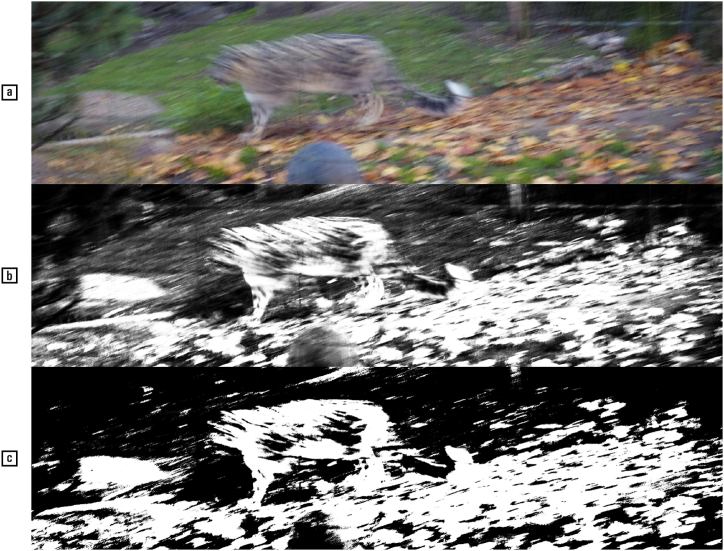
Motion perception is indispensable for detecting well-camouflaged and fast-moving targets; colour vision, however, is less helpful for tasks such as locating a snow leopard in its natural habitat. Picture (a) is in greyscale, and picture (c) is binarized, to mimic winter conditions). — Credits: Snow leopard (*Panthera uncia*), Basel Zoo, 2020; picture: Vlad Atanasiu.

By comparison, males have increased spatial awareness as opposed to vividness of object imagery [[89]: 648; [90]], more accurate depth vision [91], inter alia due to a narrower visual field [[92] (1): 106–107], and faster motion perception [41,93,94]. All these are advantages for males to catch prey and hide from ferocious beasts in the savannas of the hominids, but hardly of utility for deciphering the Rosetta Stone and other palaeographical artefacts (Fig. 17).

Considering the social specialisation of males as hunters and females as foragers, in conjunction with the opportunities to easily hide and to observe open spaces afforded by primeval human habitats, and supported by archaeological and palaeontological data, psychophysical findings such as those listed above have formed the basis of the ‘hunter–gatherer ecological hypothesis’ of the evolution of sexual dimorphisms of human vision [91,95] (but woman are also participating in hunting [96], and males are also painting in colour). This conjecture should acquire a stronger significance for the reader if we revolve the historical perspective towards implications for the present. In other words, contemporary scientists may wish to consider the impact on their interests and work methods of perpetuating traits evolved in prehistoric cultural and environmental contexts. As a practical example, the interpretation of inscriptions with low legibility could be carried out collaboratively, to profit from the complementarity of visual abilities, while image enhancement methods could be developed with personalisation in mind, just as reading glasses are not made for the average viewer [32].

### 5.2 Colour as facilitator in shape analysis

*The detection and localization of shapes in the visual field is an example of a task considerably facilitated by colour; for synaesthetes, this holds true even in the absence of physical colours.*

**Fig. 18:**
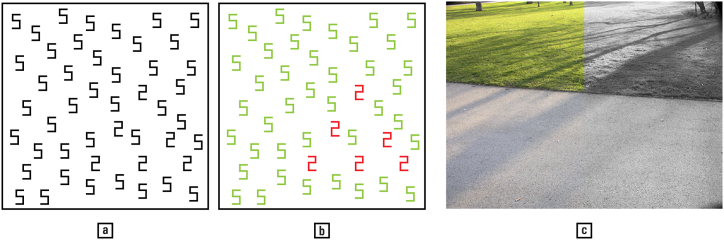
(a) A mix of ‘2’ and ‘5’ black figures, with the ‘2's arranged in a triangle. (b) Differentiation between ‘2's and ‘5's; detection of the triangle becomes much easier for both humans and machines when the figures are coloured. (c) Colour also considerably facilitates the automatic segmentation of the image into ‘grass’ and ‘pavement’ areas. — Credits: a, b. after [100]; c. Vlad Atanasiu.

During early childhood, humans display a preference for colour over shape when classifying stimuli [[97]: 150]. While this propensity diminishes with age, it does not disappear entirely. Typically, the gaze more easily detects objects with a stronger lightness contrast; at equal lightness, however, objects can appear to ‘pop out’ from their surroundings if they contrast in hue or saturation, a ‘law’ of Gestalt psychology called ‘colour saliency’ [98,99]. Fig. 18 illustrates this phenomenon, showing how shape identification and texture segmentation is influenced by colour.

The mental colouring of numbers, letters, and other shapes in the absence of physical colouring — and thus their faster classification when similarly coloured — occurs for synaesthetes, those people endowed with cross-modal perception, such as experiencing shapes as colours or sounds as flavours [101,100,102]. In the sonnet *Voyelles,* the French poet Arthur Rimbaud famously described vowels as being coloured, an example of sound-as-colour cross-modal perception: ‘black A, white E, red I, green U, blue O vowels/one day I'll tell your ineffable birth’ (it has been suggested that the order of vowels in this poem follows that in the evolution of colour naming; [103]).

### 5.3 Colour as complication in shape analysis

*In horology, the term ‘complication’ designates ‘any feature of a mechanical timepiece beyond the display of hours, minutes, and seconds’, such as a calendar, or, on certain smartwatches, heart rate [* 104 *]. By analogy, we may say that colour has many ‘complications’: it is a thicket of interacting phenomena and surprising implications. Colourblindness and subjective colours are two of the more distinctive in terms of shape perception and cognition.*

**Fig. 19:**
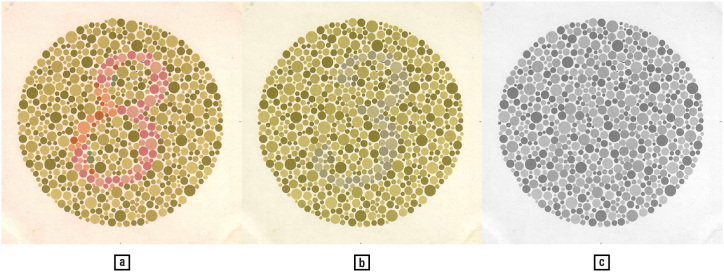
Colours create shapes — (a) Normal-sighted persons read the figure ‘8’ in this colourblindness test plate, red–green deficient persons read ‘3’, and monochromats see no shape at all. (b, c) These two plates are image processed variants of plate (a), simulating for the benefit of normal-sighted persons the red–green deficiency and the monochromatic vision, respectively; specifically, a faint ‘3’ should be seen in plate (b), and no digit in plate (c). The strength of the effect varies with the viewing distance. — Picture (b) is obtained by converting the RGB image to the L*a*b* colour space, and setting the values of the a* image channel, i.e., the green–red colour space axis, to zero, while picture (c) is obtained by applying the same procedure to the b*-channel, i.e., the blue–yellow channel, as well. The ‘3’ in picture (b) is difficult to distinguish, which illustrates that the processes involved in colourblindness are more complex than can be simulated by even a refined colour appearance model such as L*a*b*. — Credits: [Ref. [105]: plate 2].

When persons with red-green colourblindness, one form of colour deficiency of varying intensity, look at Fig. 19 a, they read the number ‘3’, while normally sighted people see an ‘8’; those who are totally colourblind see no number at all (plates of the so-called Ishihara colourblindness test: [[105]: 4]; history of colourblindess tests: [106]).

The mechanism at work in the stimuli of the so-called Ishihara tests of colourblindness, is worth our attention, since it combines at least three distinct visual effects: chromatic masking, perceptual grouping, and edge smoothing. First, the red dots belonging to the digit ‘8’, but not to the digit ‘3’, result in the same green as the background when projected on the green–red colour dimension perceived by green–red colourblind persons. Second, there exists a cluster of red dots that are common to both ‘8’ and ‘3’, whose integration forms the shape ‘8’. Third, the random size and placement of dots ensures that the joints between ‘8’ and ‘3’ are inconspicuous (similar to how typographical stochastic halftoning makes prints appear smooth, by randomizing phases in the frequency domain representation of images [107,108).

At physiological level, the phenomenon illustrated here is due to the lack of red-sensitive pigments in the eye's photoreceptor cells [[[109], [110]]: 35–37]. Depending on whether the red, green, or blue cones are affected, the condition is known as protanopia, deuteranopia, or tritanopia. Colourblindness affects about 8 % of males, and less than 1 % of women of North European ancestry, with lesser percentages in other demographics; for physiological reasons (such as the yellowing of the cornea), colour deficiencies also vary with age [[111]: 222–223; [40] (1): 388–390; [110]: 38].

The prevalence of males in computer sciences — the percentage of bachelor degrees awarded to males in computer science in the United States dropped from 85 % in 1966 to 63 % in 1984, and rebounded to 82 % in 2008 [[28]: 105–106] — has as consequence a higher average of colourblind individuals among this demographic than in the general population, a situation worth investigation for elucidating whether it is related in any way to a neglect of colour as a computational analysis feature in favour of shape. However, it can also be noted that colour-anomalous observers have the benefits of superior visual acuity and contrast sensitivity [112]. Likewise, it is yet to be determined whether the current trend in the feminisation of papyrology — about 56 % of participants at the 2019 International Congress of Papyrology were women [113] compared to only 5 % in 1937 [114] — will lead to a heightened interest in colour among scholars, and thus help advance the field.

The colourblindness ‘deficiency’ is a valuable ability for applications in steganalysis and military camouflage detection, as objects and patterns that are invisible to normal-sighted people might be detected by the colourblind [[115]; 86: 106]. There are implications for palaeographers, archaeologists, and computer scientists as well. Colourblind researchers may seek to use software packages that aid the analysis of coloured artefacts (e.g., Ref. [116]. Also, it is possible that the chromatic characteristics of artefacts change under the influence of natural or artificial factors, creating spurious shapes. It is therefore recommended that the inspection of colour images by normal-sighted persons includes the isolation of the green–red and blue–yellow channels to detect potential alterations.

The effects of colourblindness, such as revealed through the Ishihara tests, are a striking demonstration of shape identification being incontrovertibly contingent on colour. More generally, colour vision plays an important role in the construction and deconstruction of shape, as attested (with existential implications) by the use of camouflage among animals and humans [117,118,119] and a host of other perceptual processes, such as the determination of texture from colour [120]. The basic principle is that shape is determined by edges and edges by contrast, which can occur due to a difference in lightness or saturation or hue. If these examples show that colour can affect shape, the reverse is also true: shape can induce the perception of subjective colours [[92] (1): 368–369]. The detection of optical illusions [24,121] in digital imagery should thus provide computer scientists with an intellectually rewarding research field.

**Fig. 20:**
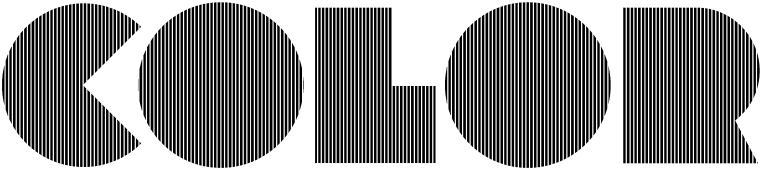
By drifting the eyes slowly over the striped texture, the observer should perceive pastel-coloured patches emerge from the black and white hatching. — Credits: composition: Vlad Atanasiu; typeface: Lot, by Svetoslav Simov.

To substantiate the well-founded nature of our entreatment, let us consider the diversity of ramifications resulting from studying the subjective colours perceivable in Fig. 20. Illusory pastel-coloured patches emerge from this achromatic hatched stimulus if the eyes drift slowly over the striped texture. Hatching is a technique used in graphic arts to simulate grey levels by means of black and white, in engraving, cartography, technical drawing, and as ‘guilloche’ interlaces (used e.g., as security features for banknotes). Such was the level of mastery attained in hatching during the Renaissance that the humanist Erasmus considered colour as almost superfluous: ‘Dürer … could express absolutely everything in monochrome, that is in black and white … And so alive is it to the eye that if you were to add colour you would spoil the effect.’ [[122]: 399] Five hundred years later, the same reticence towards colour and the belief in the ability of achromatic stimuli to simulate it — or at least to simulate the emotions colour evokes — made many great photographers and film directors wary of adopting colour film. An explicit link between hatching and colour in Western cultures is provided by its use in heraldry, where, for example, vertical hatching stands for gules (red), horizontals for azure (blue), and left diagonals for vert (green) [123,124]. Elsewhere, black-and-white patterned pottery of the pre-Columbian Mimbres Native American culture has been found to produce the same subjective colours when spun as the test disks of modern psychologists studying these so-called Fechner colours or Bentham illusion [125].

### 5.4 Challenges to benefiting from colour in shape analysis

*Colour science, with its great variety of interdependent branches, presents researchers with the concomitantly daunting and exciting challenge of becoming knowledgeable of domains outside their specializations. Two of these domains, namely historical and cultural, are discussed in the following in the context of shape analysis as impacted by the colours of writing substrates, by colour naming (the linguistic dimension of colour science), and by display hardware.*

As the Enlightenment scientist, philosopher, and novelist Margaret Cavendish once remarked, ‘no body is without colour’ [[126]: 307]; likewise, the history of script cannot be decoupled from that of the writing substrate. It suffices to consider the three-dimensionality of the majestic Roman epigraphic styles (the shadows shifting within its furrows with each passing cloud and the diurnal movement of the sun) or reflect on how, in the bureaucracies of the tenth century Middle East, handwriting styles were developed that were specific to various paper sizes [127] and, during the late twentieth century, a cornucopia of pixelated digital typefaces emerged due to the low resolution of displays and printers [128]. It would however be naive to consider colour as the primary classification criterion for substrates, as the aforementioned examples already suggest. The chromatic contrast between script and background, however important to legibility (and, thus, to the very function of writing), was often secondary to other considerations regarding script. Looking at major types of modern paper, the difference between coated and uncoated paper is one of shininess, of script-outline sharpness (due to the extent of ink absorption by the substrate), and of the velvet feel of the surface; recycled paper is not about low saturation, but about environmental issues; acid-free paper is not about the loss of whiteness, but about long-term document conservation; handmade paper has more to do with social status and nostalgia than a furtive veil of sepia colour; and the quest for increasingly white paper, observed throughout the history of Western papermaking, is interpreted as the negation of colour by desaturation and the achievement of an achromatic surface to satisfy chromophobic socio-cultural tendencies related to ideologies, race, ethnicity, class, and gender (overview: [129]; colour, computing, and ideologies: [130]; ethnography: [131]; paper colour, gender, and race: [[132]: 125–156]; early colour science and race: [[133]: 292–293]).

Human subjects research is often impacted by a lack of practical access to participants (due to e.g., financial, time, and setup complexity constraints), a limited absolute number of cases (e.g., medical conditions), and, at times, a scientific culture of complacency; this commonly results in samples of experimental participants that are too small and/or insufficiently representative, thus reducing the statistical power of the results [134]. Colour science is no exception: the foundationally important CIEXYZ 1931 ‘standard colorimetric observer’ was derived by averaging data obtained from only seventeen Britons [[135]: 189], while a formula for simulating colourblindness for the normal-sighted commonly used in computer science was developed from studies of two (sic) French people [[136]: 2650]. Furthermore, standards such as CIEXYZ 1931 colour space and the CIE 1939 definition of colour are ‘cultural developments’, guided by the input of various individuals, commissions, and national interests in addition to scientific research [[135]: 159–190]. To take an example, the reliance on lightness, saturation, and hue in contemporary colour science for defining colour spaces and studying colour naming is ‘a completely arbitrary one’, in the own words of the physiologist Johannes von Kries who was instrumental in propagating this colour model at the end of the nineteenth century [[137]: 129–132]. However, its success and continued use, are due to it being conceptually sound in terms of the physics of light and materials, and the utility of its mathematical modelling, as well as the weight of the social status of physicians and engineers within the 1930s CIE, in addition to the historical context that favoured these three colour appearance modes in Western cultures and the emergence of modern colour science within it [138,139].

Just as important as the scientific validity in colour research is the fact that human colour perception is a variable and evolving phenomenon. In this sense, for example, the study of the colour vocabulary of the ancient Greeks reveals that they were attracted not so much by hue and saturation, as in the modern Western world, but by other colour aspects such as lightness and shimmer [140,141], while the Polynesian people of the Bellona Island seem little interested in colour as an ontological category, preferring object categorization by patterns and utility [142]. More generally, it has been observed that industrialization (i.e., the complex of the chemical colour industry, colour science, standardization, and marketing) favours consistent colour naming [[143]: 10788]. Certainly, it has also contributed to augment the colourfulness of the modern world, and thereby impacting the development of colour cognition: in the span of the twentieth century the acquisition by Western children of the ability to correctly use the names of the four basic opponent colours red, blue, green, and yellow, has decreased from about eight to about four years [[75]: 149–150]. Not only are children immersed in an environment that has become chromatically richer, but toys such Lego bricks and colour crayons allow colour-focused object manipulation and contribute to develop colour as an expressive modality closely integrated with shape. Increased urbanization and intercontinental migration engender the chromatic acculturation and evolution explaining such cases as the westward spread of words for the colour ‘turqouise’ from the Middle East (with mines in the Sinai Peninsula, Iran, and Afghanistan), where it is a quintessential colour of Egyptian, Persian, and Ottoman arts (as the name implies: the ‘Turkish’ colour); as well the more frequent use of the terms ‘brown’ and ‘orange’ by Japanese migrating from countryside to Tokyo and from Japan to Germany [[79]: 141–151].

The relationship between colour and shape is also ingrained in how humans not only name objects, but think through language. Consider the metonymic use of colour terms to speak about the visibility of shapes, as in ‘pitch-black night’ (invisible shapes), ‘white nights’ (visible shapes), ‘snow white-out’ (shapeless), ‘all cats are grey at night’ (indistinct shapes), and ‘blue hour’ (ambiguous shapes) [144]. That the replacement of colour words with shape words is rarer, such as the hypothetical and poetical ‘mellow pink’ (marshmallows) and ‘sharp blue’ (ice), reveals an important aspect in the discussion of colour-based shape analysis: colour cognition has evolved in humans more recently than shape cognition [[145]: 162].

From an applications point of view (e.g., for interface design), linguistic colour categories can speed up colour differentiation, and thus potentially coloured-shape identification: light blue and dark blue targets have been classified faster by Russian speakers who have two distinct words for them, ‘sinij’ and ‘goluboj’, and no overall term for blue, than by English speakers, who possess only one basic colour term for the blue range [146,147].

Circumstances such as discussed above add to the argument that it would be misleading to apply contemporary scientific concepts of colour to the digital processing of documents from the past, or non-Western or non-English-speaking societies, or to employ a distinct linguistic, cultural, or professional group of people to label colours for machine learning when the resulting algorithm is to be used by specific, mixed, or global demographics [148]. (On colour naming, see the original study of Berlin and Kay [149], its update in Ref. [52], and the overview of [23]; on the debate on the universality versus relativity of colour cognition, see e.g., Refs. [150,151,152,153,147,154].)

Limited cultural awareness and historical knowledge are not the only impediments to benefitting from colour for shape analysis: modern colour capture, description, and display technology promote certain aspects and concepts of colours over others. A flat, light-emitting, and rigid computer display is ill-fitted to suitably reproduce the colour ‘oinops’, Homer's word for the liquid glimmer of the ocean's moving surface that evokes wine in a drinking cup [[141]; [155]: 25–40; [156]]. If our technologists shared Homer's interests, our modern colour technologies might be very different to those we have today. Instead, photographic film employed for portraits was predominantly calibrated using ‘white’ models (‘Shirleys’, or, in cinema, ‘China Girls’), meaning that other and especially darker skin tones appeared with poor shape detail and contrast with the background; this issue was not so much technical as the result of sociocultural inequalities [157,158,159].

## 6 Impact of changing social attitudes on colour use by scholars and scientists

*Having arrived at the end of this article, let us step back from the extensive technical data provided herein and conclude with a few reflections on the changing roles of colour in society and the sciences, since the social and cultural environments are co-determinant factors in how colour is perceived as an aid in shape analysis.*

A long-forgotten art-theoretical debate of the Italian Renaissance concerned the question of the primacy of shape (‘disegno’, drawing shape outlines) or colour (‘colore’, brushing to cover surfaces), which stood for meaning and affect respectively, and was decided in favour of shape [[130]: 29]. The argument has been rekindled repeatedly in the history of the arts, sciences, and technologies. Although it has been well established that Antique Greco-Roman sculptures were painted in vivid colours rather than white, museum-goers remain so conditioned to the canon of an ‘ivory’ art that they find it difficult to consider the original polychromy anything but ‘garish’ [160]. Following the invention of colour photography, its merits in respect to black and white photography were vehemently contested, with the latter retaining a more ‘noble’ status. Examples include the monochromatic work of Ansel Adams, Henri Cartier-Bresson, Richard Avedon and other great artists (in the words of Cartier-Bresson: ‘black and white transports, abstracts, and is not “normal”. Reality is a chaotic deluge […]: why then bother with colour?’ [[161]: 155–156]); consider also the continued production of monochromatic cameras for art photography, such as the high-end digital Monochrom Leica (its marketing slogan: ‘Discover more in black and white’). Reflecting recently on the status of colour studies, the eminent French historian of colour Michel Pastoureau still considers that ‘when you are a historian of painting, focusing your academic work on the issue of colour is handicapping yourself, at least in France’ [162] — as if the terms *history* and *colour* are antithetical, as if we tend to think of the past as a realm of Piranesian grayscale shadows, a new achromatic trick of human memory.

These examples of chromophobia in Western societies across time and place also have their manifestations in the sciences, notwithstanding Goethe, who valued the results of his research on colour more highly than his entire literary output [[21]: 115–119]. In respect to the pace of scientific progress, topics of interest, and the scientists' attitudes, it has been noted that ‘the quantitative measurement of light intensity was not commonplace until the 1930s […] long after quantification had become central to other fields of science’, [[135]: 3], while until the end of the twentieth century, colour was secondary to the study of shape in psychology, with many psychophysical experiments still being conducted on greyscale stimuli [[24]: xx]. In medical radiography, black-and-white reproduction remains ubiquitous, despite the presence of alternatives (blue-tinted X-ray films have been commercially available since 1933 [[25]: 138]). The dominant ‘Colourbook theory’ considered colour as almost a frivolous act of filling in the space between edges after the all-important shape segmentation had been completed by more fundamental, and evolutionarily older, neural processes, causing one researcher to exclaim in frustration: ‘Colours are surely not just ornamental baubles, installed in us by Providence to soften the harsh realities of life and give employment to painters, interior decorators and crayon manufacturers’ [[23]: 371]. Colours were also seen as properties of objects, as opposed to the anti-realist perspective, according to which they are constructs of the brain, and thus not reducible to purely physical investigation. Because ‘human factors’ are inherently multifactorial, and difficult to measure, reproduce, and automate, this dichotomy was a source of dissension between psychologists and physicists during the early CIE standardisation work on colorimetry in the 1930s [[135]: 130–132]. In respect to the cognitive paradigms of another discipline, you can do mathematics with shapes, but hardly with colours (geometry and arithmetic are the oldest areas of mathematics, while the more limited-in-scope chromatic graph theory emerged only during the nineteenth century; interestingly, it did so as a solution domain for solving the shape-related mapmaking problem known as the Four-Colour Problem [1852]. This was contemporary with the fascinating work by Oliver Byrne, a British surveyor, on representing the demonstrations in Euclid's *Elements* by means of coloured diagrams [9]; the connexion of these events to cartography indicate a discipline that entertains a traditionally strong interest in using the interaction of shape and colour as representation modalities, with the refinement of shape-from-texture-and-colour techniques for simulating topography by relief shading being one example [163,66,164].).

The social upheavals of the 1960s were accompanied by a firework of colours, from ‘flower power’ politics to the psychedelic colour worlds produced by drug consumption, no doubt in conjunction with technologies such as colour photography, cinema, and television [130]. Even the Apollo missions to the moon generated interest in colour and shape perception, as astronauts reported the lunar exterior to be ‘a grey surface modified by weak-to-strong overtones of green, brown, or blue’, quite difficult to describe and very different from our concept of an achromatic environment, an otherworldly experience further enhanced by the extreme sharp details up to the horizon enabled by the absence of atmosphere [68].

Soon, the sciences took this ‘colour turn’ themselves: with the formulation of quantum chromodynamics in the 1970s, physicists began to use trichromatic additive and subtractive colours as a ‘chromogrammatic’ tool to think and communicate by words and colouring about the interaction between quarks and gluons (to the regrets of Richard Feynman, who had preferred ‘wonderful Greek names’ [[79]: 3]), while signal processing engineers had an established and growing chromatic naming tradition for noise types – attested since 1943, ‘white noise’ has since entered common language (tellingly, by relating colour to noise, colour is again construed as something undesirable) [[[165], [166], [167]]: ‘white noise’]. A veritable ‘colourmania’ took hold of scientists attracted to chromatic colourmaps, such as the ‘rainbow’ one, when colour displays and graphic cards became widely available. Colour also permeated popular science: weren't the kids of the 1980s fascinated by growing multicolour fractals on their home computers and squinting their eyes at colourful random-dot stereograms? In psychology, research shifted from the study of colour and shape in isolation to their integration. The theories of embodiment (i.e., the mind is shaped by the specificities of a given body), enaction (i.e., the mind is shaped by interaction with the environment), and ecology (i.e., perception and cognition are predictively driven by statistics of various aspects of the environment) converged to give rise to new theories of colour, such as adverbialism, which posits that colour is the manifestation of interactions between environment, body, and mind [[[168], [169]]: 131–158]. The psychophysics of low-level vision is identifying an increasingly long list of phenomena attesting to the utility of colour per se and its interaction with shape and other representations of the world [19]. In computer science, too, colour has become since some time already routinely used in conjunction with other features; for example, researchers proposed twenty years ago the integration of object colour, shape, and semantics for the navigation of digital image libraries [170], an approach subsequently implemented in general public applications such as Google Image, which offers a search-by-colour feature. Such developments must be appreciated within the context of the broader trend towards integrative technologies, e.g., ensemble methods that fuse the classification results obtained by different methods [171], and multibiometrics, which integrates evidence across sensors and modalities [172].

Summing up the trends described above, we observe an increase in the performance of humans in both biological-based and technological-assisted extraction of colour information from light, in the natural environment for survival (ripe fruits), and in the human-made environment for social advantage in terms of status (the imperial purple and the blue jeans) and information management capabilities (fluorescent highlighters and digital colourmaps).

The practice of script analysis by humans and machines, however, remains dominated by a focus on shape and an almost pathological rejection of colour, as exemplified by the systematic monochromatic reproduction of script samples whenever it is possible to discard the background, as well as by the binarization of images prior to computational analysis. Time and accidents, however, have ensured that real documents are seldom perfectly achromatic, and that even otherwise achromatic digital documents may contain unintended colour pixels. Besides the historical and forensic information that these colour artefacts represent, they also provide additional and useful information supporting the goals of preprocessing and script analysis. This article has presented selected case studies from along the chain of image formation, with the goal of convincing the reader of the utility of colour for shape analysis. Encouragingly, we already see numerous indicators to suggest increasing interest in colour among the disciplines involved in one way or another with script and other shapes. As a case in point, even typography, the ‘black art’, today wears a brighter and more colourful face, aided by a modern digital technology known as ‘’ [71,173].

## 7 Conclusions

Considering, on the one hand, the lack of familiarity with colour science among scholars and computer scientists and, on the other hand, the potential of colour for shape analysis, this article has endeavoured to improve the state of colour science literacy by raising awareness of its benefits and contributing a ‘nutshell’ introduction to colour science, which is illustrated with case studies from the domain of script analysis and covers management, computational, psychological, and social colour aspects.

While each section title and introductory paragraph points toward specific rationales for colour-based shape analysis, we summarize here the main lessons:

· there exists a need to improve colour science literacy;
· colour has many potential benefits for analysing shapes;
· colour science is complex, constituted of many interacting disciplines;
· data fidelity in reproducing, formatting, converting, and displaying images, along with task-oriented solutions, are necessary for benefitting from colour;
· even simple computational methods are helpful in a variety of applications;
· psychological factors in colour processing ought not to be neglected and can be beneficially leveraged, often demanding only limited resources;
· sociocultural attitudes to colour unobtrusively influence its use.

Looking up from script towards the stars, astronomers give us a persuasive ultimate rationale for the utility of colour images, referring to the inference of star temperature from their colour: ‘colour is knowledge’ [[174]: 34].

## Funding sources

This work was supported by the 10.13039/100000001Swiss National Science Foundation, project n◦ PZ00P1_174149, ‘Reuniting fragments, identifying scribes and characterizing scripts: the digital palaeography of Greek and Coptic papyri’.

## Data availability statement

No data was used for the research described in this article.

## CRediT authorship contribution statement

**Vlad Atanasiu:** Conceptualization, Data curation, Formal analysis, Funding acquisition, Investigation, Methodology, Project administration, Resources, Software, Supervision, Validation, Visualization, Writing – original draft, Writing – review & editing. **Peter Fornaro:** Conceptualization, Data curation, Formal analysis, Investigation, Methodology, Resources, Software, Validation, Visualization, Writing – review & editing.

## Declaration of competing interest

The authors declare that they have no known competing financial interests or personal relationships that could have appeared to influence the work reported in this paper.

## Footnotes

∗ One recurring example for the need of colour science literacy can be gathered from articles on image processing, were sometimes the RGB and HSV colour spaces are used, while perceptually uniform spaces, such as CIELAB, would be more appropriate, for example, to convert an image to grayscale or manipulate the chromatic channels. A further (notorious) example is the use of the ‘rainbow’ colour map, which introduces spurious perceptual classes in otherwise monotonic linear data, and misleading image interpretation by showing shapes where there are none [36]. That ignorance of such trivial colour facts persists among scientists even decades after the issues were stated and remedies developed, indicates that colour literacy remains of actuality. (On colourmap misuses among scientists see Ref. [175]; on image processing for papyrology see [120: 94]; on the wanting state of colour literacy among visual artists, designers, and architects see Ref. [27]; the Color Literacy Project is an advocacy group from the sciences, arts, and the industry focused on promoting colour education in the fields of sciences, technology, engineering, arts, and mathematics (STEAM) [176]; the International Colour Day takes place annually on March 21; it was established by the International Colour Association (AIC) to promote colour science awareness [177].).

## References

[1] Déroche F., with Berthier A., Guesdon M.-G., Guineau B., Richard F., Vernay-Nouri A., Vezin J., Waley M.I. (2006).

[2] Fu Sh, Lowry G.D., Yonemura A. (1986).

[3] Massoudy H. (1991).

[4] Cendrars B. (2009).

[5] Le Rider J. (1997).

[6] Ripoll É. (2018).

[7] Sauer Th (2009).

[8] Caraës M.-H., Marchand-Zanartu N. (2011).

[9] Byrne O. (1847). https://archive.org/details/firstsixbooksofe00byrn.

[10] Atanasiu V. (2000). Le Retroencrage: analyse du ductus des écritures d’après le dégradé du coloris de l’encre. Gazette du Livre Medieval.

[11] Renesse R. L. van (2005).

[12] Rodney A. (2005).

[13] Giorgianni E.J., Madden Th E. (2008).

[14] Sharma A. (2018).

[15] Green Ph (2010). Color Management: Understanding and Using ICC Profiles.

[16] Poynton Ch (1998).

[17] Berns R.S. (2019).

[18] Reinhard E., Khan E.A., Akyüz A.O., Johnson G. (2008).

[19] Shevell S.K. (2003). The Science of Color.

[20] Wyszecki G., Stiles W.S. (2000).

[21] Crone R.A. (1999.).

[22] Hoffman D.D. (2008). Conscious realism and the mind-body problem. Mind Matter.

[23] Hardin C.L. (1988).

[24] Shapiro A., Todorović D. (2017). The Oxford Compendium of Visual Illusions.

[25] Curry T.S., Dowdey J.E., Murry R.C. (1990).

[26] Wikimedia Commons Contributors (2023). https://commons.wikimedia.org/wiki/File:Wiki_dell_lcd.jpg.

[27] Hirschler R., Csillag P., Manyé P., Neder M., How much colour science is not too much?, Color Ressearch & Application 43 (2018) 977–992, doi:10.1002/col.22275. (Critique of colour literacy among visual artists, designers, and architects; discusses misconceptions about colour, and presents the fundamentals for a colour science curriculum for non-scientists.).

[28] Atanasiu V. (2014).

[29] [ FADGI] United States Government (2022). http://www.digitizationguidelines.gov.

[30] [ Metamorfoze] National Library of the Netherlands (2022). https://www.metamorfoze.nl.

[31] Van Poucke S., Vander Haeghen Y., Vissers K., Meert Th, Jorens Ph (2010). Automatic colorimetric calibration of human wounds. BMC Med. Imag..

[32] Baba N., Isobe S., Norimoto Y., Noguchi M. (1985). Stellar speckle image reconstruction by the shift-and-add method. Appl. Opt..

[33] Raskar R., Nielsen F. (2009). Emerging Trends in Visual Computing: LIX Fall Colloquium, ETVC 2008, Palaiseau, France, November 18-20, 2008, Revised Selected and Invited Papers.

[34] Yuen M., Wu H.R., Rao K.R. (2005). Digital Video Image Quality and Perceptual Coding.

[35] Pennebaker W.B., Mitchell J.L. (1993).

[36] Rogowitz B.E., Treinish L.A. (1998). Data visualization: the end of the rainbow. IEEE Spectrum.

[37] Fairchild M.D. (2013).

[38] Kuehni R.G., Schwarz A. (2008).

[39] Stockman A., Brainard D.H., Elliot A.J., Fairchild M.D., Franklin A. (2015). Handbook of Color Psychology.

[40] Poynton Ch (2002).

[41] Abramov I., Gordon J., Feldman O., Chavarga A. (2012). Sex & vision I: spatio-temporal resolution. Biology of sex differences. Biol. Sex Differ..

[42] Abramov, I., Gordon, J., Feldman, O., and Chavarga, A. Sex and vision II: Color appearance of monochromatic lights, *Biol. Sex Differ.* 3 (2012) article 21, doi:10.1186/2042-6410-3-21. **(On sexual differences in human vision.)**. 10.1186/2042-6410-3-21 PMC3483194 22943488

[43] Morovič J. (2008).

[44] Zuiderveld K., Heckbert P.S. (1994). Graphic Gems IV.

[45] Atanasiu V., Marthot-Santaniello I. (2021). Personalizing image enhancement for critical visual tasks: improved legibility of papyri using color processing and visual illusions. Int. J. Doc. Anal. Recogn..

[46] Land E.H. (1977). The retinex theory of color vision. Sci. Am..

[47] McCann J.J. (2017). Retinex at 50: color theory and spatial algorithms, a review. J. Electron. Imag..

[48] Jobson D.J., Rahman Z.-ur, Woodell G.A. (1997). A multiscale retinex for bridging the gap between color images and the human observation of scenes. IEEE Trans. Image Process..

[49] (2017). Special section on retinex at 50. J. Electron. Imag..

[50] Foster D.H., Amano K., Nascimento S.M.C., Foster M.J. (2006). Frequency of metamerism in natural scenes. J. Optical Soc.Am. A, Optics, Image Sci. Vision.

[51] Hariharan H., Koschan A., Abidi B., Gribok A., Abidi M. (2006). Fusion of visible and infrared images using empirical mode decomposition to improve face recognition. Proceedings of the IEEE 2006 International Conference on Image Processing (ICIP 2006), 8–11 October 2006, Atlanta, GA, USA,.

[52] Kay P., Maffi L. (1999). Color appearance and the emergence and evolution of basic color lexicons. Am. Anthropol..

[53] Gillespie A.R., Kahle A.B., Walker R.E. (1986). Color enhancement of highly correlated images. I. Decorrelation and HSI contrast stretches. Rem. Sens. Environ..

[54] Gillespie A.R., Kahle A.B., Walker R.E., Erratum A.R., Kahle A.B., Walker R.E. (1986). Color enhancement of highly correlated images. I. Decorrelation and HSI contrast stretches, remote sens. Environ. 20: 209–235. Rem. Sens. Environ..

[55] Gillespie A.R., Kahle A.B., Walker R.E. (1987). Color enhancement of highly correlated images. II. Channel ratio and “chromaticity” transformation techniques. Rem. Sens. Environ..

[56] Pratikakis I., Zagoris K., Karagiannis X., Tsochatzidis L., Mondal T., Marthot-Santaniello I. (2019).

[57] Van de Weijer J., Gevers Th, Smeulders A.W. (2006). Robust photometric invariant features from the color tensor. IEEE Trans. Image Process..

[58] Chen J., Pappas T.N., Mojsilovic A., Rogowitz B.E. (2005). Adaptive perceptual color-texture image segmentation. IEEE Trans. Image Process..

[59] Canny J. (1986). A computational approach to edge detection. IEEE Trans. Pattern Anal. Mach. Intell..

[60] Nobuyuki Otsu (1979). Auto thresholding. IEEE Transactions on Systems, Man, and Cybernetics, SMC-.

[61] Gevers Th, Gijsenij A., van de Weijer J., Geusebroek J.-M. (2012).

[62] Kingdom F.A.A., Beauce C., Hunter L. (2004). Colour vision brings clarity to shadows. Perception.

[63] Breuil C., Jennings B.J., Barthelme S., Guyader N., Kingdom F.A.A. (2019). Color improves edge classification in human vision. PLoS Comput. Biol..

[64] Ebner M. (2007.).

[65] Campenhausen C. von (1986). Photoreceptors, lightness constancy and color vision. Naturwissenschaften.

[66] Collier P., Forrest D., Pearson A. (2003). The representation of topographic information on maps: the depiction of relief. Cartogr. J..

[67] Jones D., Amaral M. (2019).

[68] Saunders A. (2022).

[69] Keplinger Th (2020). https://www.worteimdunkel.at/?p=6713.

[70] Russ J.C., Neal F.B. (2016).

[71] L'Excellent Th (2009). https://typo.thomaslexcellent.com.

[72] Chandler D.M. (2013). Seven challenges in image quality assessment: past, present, and future research. Int. Scholarly Res. Notices (ISRN) Signal Process..

[73] Beghdadi A., Larabi M.-C., Bouzerdoum A., Iftekharuddin K.M. (2013). A survey of perceptual image processing methods. Signal Process. Image Commun..

[74] Le Moan S., Farup I., Blahová J. (2018). Towards exploiting change blindness for image processing. J. Vis. Commun. Image Represent..

[75] Davidoff J. (1991).

[76] Land M.F., Nilsson D.-E. (2012).

[77] Jacobs G.H., Elliot A.J., Fairchild M.D., Franklin A. (2015). Handbook of Color Psychology.

[78] Kawamura Sh, Hiramatsu Ch, Melin A.D., Schaffner C.M., Aureli F., Fedigan L.M., Hirai H., Imai H., Go Y. (2012). Post-genome Biology of Primates.

[79] Zollinger H. (1999).

[80] Shepard R.N., Barkow J., Cosmides L., Tooby J. (1992). The Adapted Mind: Evolutionary Psychology and the Generation of Culture.

[81] Regan B.C., Julliot C., Simmen B., Viénot F., Charles-Dominique P., Mollon J.D. (2001). Fruits, foliage and the evolution of primate colour vision. Phil. Trans.: Biol. Sci..

[82] Sumner P., Mollon J.D. (2000). Catarrhine photopigments are optimized for detecting targets against a foliage background. J. Exp. Biol..

[83] Jacobs G.H. (1992). Ultraviolet vision in vertebrates. Am. Zool..

[84] Dolder R., Counsell J.N. (1981). Natural Colours for Food and Other Uses.

[85] Wright L., Black F. (2013). Monochrome males and colorful females: do gender and age influence the color and content of drawings?. Sage Open.

[86] Sjoberg E.A., Wilner R.G., D'Souza A., Cole G.G. (2023). The stroop task sex difference: evolved inhibition or color naming?. Arch. Sex. Behav..

[87] Webster M.A., Elliot A.J., Fairchild M.D., Franklin A. (2015). Handbook of Color Psychology.

[88] Jameson K.A., Winkler A.D., Goldfarb K. (2016).

[89] Blajenkova O., Kozhevnikov M. (2009). The new object-spatial-verbal cognitive style model: theory and measurement. Appl. Cognit. Psychol..

[90] Voyer D., Voyer S., Bryden M.P. (1995). Magnitude of sex differences in spatial abilities: a meta-analysis and consideration of critical variables. Psychol. Bull..

[91] Sanders G., Sinclair K., Walsh T. (2007). Testing predictions from the hunter-gatherer hypothesis — 2: sex Differences in the visual processing of near and far space. Evol. Psychol..

[92] Boff K.R., Lincoln J.E. (1988). *Engineering Data Compendium: Human Perception and Performance*.

[93] Murray S.O., Schallmo M.-P., Kolodny T., Millin R., Kale A., Thomas Ph, Rammsayer T.H., Troche S.J., Bernier R.A., Tadin D. (2018). Sex differences in visual motion processing. Curr. Biol..

[94] Shaqiri A., Roinishvili M., Grzeczkowski L., Chkonia E., Pilz K., Mohr, Brand A., Kunchulia M., Herzog M.H. (2018). Sex-related differences in vision are heterogeneous. Sci. Rep..

[95] Silverman I., Eals M., Barkow J., Cosmides L., Tooby J. (1992). The Adapted Mind: Evolutionary Psychology and the Generation of Culture.

[96] Anderson A., Chilczuk S., Nelson K., Ruther R., Wall-Scheffler C. (2023). The Myth of Man the Hunter: women's contribution to the hunt across ethnographic contexts. PLoS One.

[97] Bornstein M.H., Elliot A.J., Fairchild M.D., Franklin A. (2015). *Handbook of Color Psychology*.

[98] Folk Ch L., Elliot A.J., Fairchild M.D., Franklin A. (2015). Handbook of Color Psychology.

[99] Metzger W. (2006).

[100] Ramachandran V.S., Hubbard E.M. (2001). Synaesthesia — a window into perception, thought and language. J. Conscious. Stud..

[101] Cytowic R.E., Eagleman D.M. (2009).

[102] Robertson L.C., Sagiv N. (2005). SYNESTHESIA: Perspectives from Cognitive Neuroscience.

[103] Ginsburgh V., Metzidakis S. (2019). On rimbaud's "vowels," again: vowels or colors?. Athens J. Philol..

[104] Contributors Wikipedia (2023). https://en.wikipedia.org/wiki/Complication_(horology.

[105] Ishihara Sh (1972).

[106] French A., Rose K., Thompson K., Cornell E. (2008). The Evolution of Colour Vision Testing. Australian Orthoptic Journal.

[107] Kovesi P. (2003). Phase congruency detects corners and edges. Proceedings of the Seventh International Conference on Digital Image Computing: Techniques and Applications.

[108] Lau D.L., Arce G.R. (2001).

[109] Elliot A.J., Fairchild M.D., Franklin A. (2015). Handbook of Color Psychology.

[110] McIntyre D. (2002).

[111] Parry N.R.A., Elliot A.J., Fairchild M.D., Franklin A. (2015). Handbook of Color Psychology.

[112] Doron R., Sterkin A., Fried M., Yehezkel O., Lev M., Belkin M., Lev M., Belkin M., Rosner M., Solomon A.S., Mandel Y., Polat U. (2019). Spatial visual function in anomalous trichromats: is less more?. PLoS One.

[113] Capasso M., Davoli P. (2019). *Abstracts of the 29th International Congress of Papyrology, 28 July – 8 August 2019, Lecce, Italy*.

[114] Allberry C.R.C. (1938).

[115] Morgan M.J., Adam A., Mollon J.D. (1992). Dichromats detect colour-camouflaged objects that are not detected by trichromats. Proc. Biol. Sci..

[116] Farup I. (2020). Individualised halo-free gradient-domain colour image daltonisation. J. Imaging.

[117] Behrens R.R. (2002).

[118] Ruxton G.D., Allen W.L., Sherratt Th N., Speed M.P. (2019).

[119] Stevens M., Merilaita S. (2011).

[120] Kingdom F.A.A. (2003). Color brings relief to human vision. Nat. Neurosci..

[121] Wade N.J. (2005).

[122] Erasmus D., Sowards J.K. (1985). De Pueris Instituendis, De Recta Pronuntiatione.

[123] Contributors Wikipedia (2023). https://en.wikipedia.org/wiki/Hatching_(heraldry.

[124] Contributors Wikipedia (2023). Schraffur [de]. *Wikipedia*. https://de.wikipedia.org/wiki/Schraffur.

[125] Whittlesey S.M., Subjective color in Mimbres black-on-white pottery, KIVA: J. Southwestern Anthropology and History 80 (1) (2014) 45–70, doi:10.1179/0023194015z.00000000039. (Making colour with black and white.).

[126] Chamberlain C. (2019). Color in a material world: Margaret cavendish against the early modern mechanists. Phil. Rev..

[127] Atanasiu V. (2004). Les réalités subjectives d’un paléographe arabe du X^e^ siècle. Gazette du Livre Medieval.

[128] Omagari T. (2019).

[129] Pastoureau M. (2008).

[130] Kane C.L. (2014).

[131] Taussig M. (2009).

[132] Senchyne J. (2020).

[133] Taylor G. (2005).

[134] Brysbaert M. (2019). How many participants do we have to include in properly powered experiments? A tutorial of power analysis with reference tables. J. Cognition.

[135] Johnston S.F. (2001).

[136] Brettel H., Viénot F., Mollon J.D. (1997). Computerized simulation of color appearance for dichromats. J. Opt. Soc. Am..

[137] Mausfeld R., Saunders B., van Brakel J. (2002). Theories, Technologies, Instrumentalities of Color: Anthropological and Historiographic Perspectives.

[138] Decock L., Saunders B., van Brakel J. (2002). *Theories,**Technologies*, *Instrumentalities of Color: Anthropological and Historiographic Perspectives*.

[139] Van Brakel J., Saunders B., van Brakel J. (2002). Theories, Technologies, Instrumentalities of Color: Anthropological and Historiographic Perspectives.

[140] Gage J. (1993).

[141] Sassi M.M. (2017). https://aeon.co/essays/can-we-hope-to-understand-how-the-greeks-saw-their-world.

[142] Kuschel R., Monberg T. (1974). ‘We don't talk much about colour here’: a study of colour semantics on Bellona Island. Man.

[143] Gibson E., Futrell R., Jara-Ettinger J., Mahowald K., Bergen L., Ratnasingam S., Gibson M., Piantadosi S.T., Conway B.R. (2017). Color naming across languages reflects color use. Proc. Natl. Acad. Sci. USA.

[144] Strik Lievers F., Huang Ch-R., Xiong J., Wen X., Taylor J.R. (2021). The Routledge Handbook of Cognitive Linguistics.

[145] Zollinger H., Rentschler I., Herzberger B., Epstein D. (1988). Beauty and the Brain: Biological Aspects of Aesthetics.

[146] Paramei G.V. (2005). Singing the Russian blues: an argument for culturally basic color terms. Cross Cult. Res..

[147] Winawer J., Witthoft N., Frank M.C., Wu L., Wade A.R., Boroditsky L. (2007). Russian blues reveal effects of language on color discrimination. Proc. Natl. Acad. Sci. USA.

[148] Xiang Ch (2022). https://www.vice.com/en/article/wxnaqz/ai-isnt-artificial-or-intelligent.

[149] Berlin B., Kay P. (1969).

[150] Abbott J.T., Griffiths T.L., Regier (2016). Focal colors across languages are representative members of color categories. Proc. Natl. Acad. Sci. USA.

[151] Lindsey D.T., Brown A.M., Brainard D.H., Apicella C.L. (2015). Hunter-gatherer color naming provides new insight into the evolution of color terms. Curr. Biol..

[152] Roberson D., Davidoff J., Davies I.R.L., Shapiro L.R. (2005). Color categories: evidence for the cultural relativity hypothesis?. Cognit. Psychol..

[153] Saunders B.A.C., van Brakel J. (1997). Are there nontrivial constraints on colour categorization?. Behav. Brain Sci..

[154] Witzel Ch (2016). New insights into the evolution of color terms or an effect of saturation?. i-Perception.

[155] Deutscher G. (2010).

[156] Fairchild M.D. (2006). The colors of wine. Int. J. Wine Res..

[157] Harding X., Keeping ‘Insecure’ Lit: HBO Cinematographer Ava Berkofsky on Properly Lighting Black Faces; MIC, June 9, 2017. https://www.mic.com/articles/184244/keeping-insecure-lit-hbo-cinematographer-ava-berkofsky-on-properly-lighting-black-faces. (Accessed 4 September 2023).

[158] Roth L. (2009). Looking at shirley, the ultimate norm: colour balance, image technologies, and cognitive equity. Can. J. Commun..

[159] Yue G. (2015).

[160] Brinkmann V., Dreyfus R., Kock-Brinkmann U. (2017). *Gods in Color: Polychromy in the Ancient World*.

[161] Guerrin M. (2008).

[162] Broué C., Pastoureau M. (2023). https://www.radiofrance.fr/franceculture/podcasts/a-voix-nue/la-couleur-cet-unique-et-insaisissable-objet-1890712.

[163] Ammann L., Barla P., Granier X., Guennebaud G., Reuter P. (2012). Surface relief analysis for illustrative shading. Comput. Graph. Forum.

[164] Hurni L. (2008).

[165] Bock von Wülfingen B. (2019). *Science in Color: Visualizing Achromatic Knowledge*.

[166] OED = *Oxford English dictionary*. https://www.oed.com.

[167] Wikipedia Contributors (2023). https://en.wikipedia.org/wiki/Colors_of_noise.

[168] Chirimuuta M. (2015).

[169] Chirimuuta M., Kingdom F.A.A., The uses of colour vision: ornamental, practical, and theoretical, Minds Mach. 25 (2015) 213–229, doi:10.1007/s11023-015-9364-z. (On the interaction of colour and shape; see also the other publications of author Kingdom.).

[170] Mojsilović A., Gomes J., Rogowitz B. (2002). ISee: perceptual features for image library navigation. Proceedings of SPIE Conference on Human Vision and Electronic Imaging.

[171] Kuncheva L.I. (2014).

[172] Ross A.A., Nandakumar K., Jain A.K. (2006).

[173] Van Wageningen M. (2019).

[174] Rector T.A., Arcand K., Watzke M. (2015).

[175] Crameri F., Shephard G.E., Heron P.J. (2020). The misuse of colour in science communication. Nat. Commun..

[176] [CLP] Color literacy project. 2023. https://colourliteracy.org.gov. (Accessed 23 January 2023).

[177] Contributors Wikipedia (2023). https://en.wikipedia.org/wiki/International_Colour_Day.

